# Lessons learned while exploring the impact of movement-tracking feedback on the experiences of children with neuromotor disorders taking part in interactive home exercise programs: a multi-case mixed methods study

**DOI:** 10.1186/s12984-025-01819-1

**Published:** 2026-02-27

**Authors:** Marina Petrevska, F. Virginia Wright, Ajmal Khan, Selvi Sert, Ilana Ferreira, Sarah Munce, Darcy Fehlings, Elaine Biddiss

**Affiliations:** 1 Holland Bloorview Kids Rehabilitation Hospital, Bloorview Research Institute, 150 Kilgour Road, Toronto, ON M4G 1R8 Canada; 2 Rehabilitation Sciences Institute, Temerty Faculty of Medicine, University of Toronto, Toronto, ON Canada; 3 Department of Physical Therapy, Temerty Faculty of Medicine, University of Toronto, Toronto, ON Canada; 4 Toronto Rehabilitation Institute, KITE, University Health Network, Toronto, ON Canada; 5 Department of Paediatrics, Temerty Faculty of Medicine, University of Toronto, Toronto, ON Canada; 6 Institute of Biomedical Engineering, Temerty Faculty of Medicine, University of Toronto, Toronto, ON Canada

**Keywords:** Cerebral palsy (CP), Exercise (exercise therapy), Home-based rehabilitation, Virtual applications, Virtual reality, Gaming, Mixed methods, Design thinking

## Abstract

**Background:**

Home exercise programs prescribed to children with cerebral palsy (CP) are often associated with low adherence. Interactive technologies can help motivate and guide children through exercise programs at home, reducing onus on parents. This study sought to understand the impact of movement-tracking feedback on children’s engagement and parents’ experiences within an interactive computer play home exercise program (ICP-HEP), Bootle Boot Camp.

**Methods:**

A multi-case mixed methods study was conducted with three children with CP and their parents. In the quantitative single case experimental design with alternating treatments phase, children used the ICP-HEP with and without movement-tracking feedback for four weeks, and exercise adherence, exercise fidelity (movement performance quality), perceived level of fun and helpfulness for the body (i.e., 5-point rating scales and survey) were evaluated. The version (feedback/no feedback) with the highest exercise adherence was carried out for two additional weeks. Dyadic (child/parent) qualitative interviews followed. Quantitative data were analyzed using visual and statistical approaches. Qualitative data were analyzed using directed content analysis. Quantitative and qualitative results were merged through narrative weaving and joint displays.

**Results:**

Accuracy of the movement tracking and feedback provided varied among children, exercises, and play environments. Feedback may have contributed positively to exercise adherence for two children, with a significant enhancement (*p* < 0.001) for one of these children, and no observed negative impacts for the third child. Parents and one child perceived feedback as generally being useful for learning about movement quality, however when perceived to be inaccurate, it may have been ignored. While children had varied perspectives on how fun and helpful feedback was, it was valued by all parents. All children experienced some frustration due to sporadic technical issues. All children/parents preferred Bootle Boot Camp over conventional home programs, and suggested game refinements to enhance this ICP-HEP experience.

**Conclusion:**

Use of an interactive therapy game has the potential to support children’s adherence to and children’s/parents’ experiences with home exercise, with feedback impacting children differently based on personal and environmental factors. This study serves as a foundation for future game refinements and larger-scale testing that will continue to explore the impact of feedback within an ICP-HEP.

*Trial registration*: NCT05998239.

**Supplementary Information:**

The online version contains supplementary material available at 10.1186/s12984-025-01819-1.

## Introduction

Cerebral palsy (CP) is the most common cause of childhood-onset physical disability, resulting in muscle weakness, balance deficits, spasticity, and reduced muscle control [1, 2]. Clinicians often prescribe home exercise programs (HEPs) to clients with CP and other neuromotor disorders to promote strengthening, flexibility, endurance and motor learning [3–5]. Goal-directed HEPs have been recognized as an effective intervention in CP [6, 7], with home-based training helping to improve standing, walking, running, and jumping [4]. HEPs offer practical advantages to families by eliminating travel and financial constraints, while enabling flexibility around other planned activities [4, 8]. Challenges of HEPs include maintaining families’ engagement and ensuring that exercises are completed as prescribed (i.e., exercise fidelity) [9]. Parents have an important role in supporting HEPs, which can lead to increased pressures on the parent that may negatively impact the parent-child relationship [5, 9]. 

Client engagement is a key factor in the success of HEPs and describes a motivational commitment to the treatment process encompassing behavioural (active participation, adherence, exercise fidelity), affective (emotional involvement) and cognitive (beliefs that the intervention will be successful) components [10]. Personal factors which have been shown to positively influence the adherence of young people with CP to HEPs include promotion of autonomy, belief that the program will lead to meaningful changes, and effective time management skills to balance competing interests [11]. Family supports (e.g., encouragement, direction on how to perform exercises), use of an exercise logbook (to document number of exercise repetitions and sets), short exercise programs, and physiotherapist (PT) support have further been shown to contribute to program adherence [11]. 

Previous research has identified the need for a therapy prescription application developed for children with neuromotor disorders to help them engage in individualized HEPs [3]. Caregivers and therapists have also described a need for HEP monitoring and greater individualized feedback to optimize movement performance and prevent compensatory patterns that could limit therapeutic efficacy [12–15]. While movement quality has been recognized as an important aspect of exercise, it is challenging to quantify across diverse children and individualized HEPs [3, 16]. This emphasis on movement performance is particularly pertinent to reduce potential injuries during technology-supported interventions, with injuries related to overuse and poor movement quality previously cited in the literature when commercial, entertainment-based systems, such as the Nintendo Wii, are used [17]. 

These challenges led to the conceptualization and development of a novel interactive computer play home exercise program (ICP-HEP), Bootle Boot Camp, that enables home program prescription and delivery in a gamified format with movement feedback provided via 3D body tracking to promote safe, engaging and high-quality movement performance [18]. Extensive informal testing among the design team and knowledge holders was conducted over a one-year period [18] prior to formal lab-based testing with five neurotypical and two neurodiverse children [19]. This lab-based testing revealed general acceptance of the ICP-HEP [18] and good-to-excellent tracking accuracy for a range of exercises relative to the gold standard motion analysis system [19]. Specifically, exercises such as sit to stand and seated star jump demonstrated F_1_ scores (i.e., the harmonic mean of precision and recall, ranging from 0 [low] to 1 [perfect precision and recall]) over 90%, hip abduction and kicking between 80 and 90%, and exercises such as hip flexion, lateral step, and backwards step, ranging from 70–80% [19].

The overall mixed methods objective of this study was to understand the impact of Bootle Boot Camp’s movement-tracking feedback on the behavioural, affective and cognitive engagement outcomes and home program experiences of children with CP and other neuromotor disorders, aged 6–14 years, Gross Motor Function Classification System levels I–II [20], and their parents. Findings are intended to inform Bootle Boot Camp next stage development/refinement and implementation, while providing recommendations for future ICP evaluation and translation.

Quantitative objectives were to:

1. Compare children’s behavioural engagement, primarily exercise adherence (i.e., proportion of prescribed exercise repetitions attempted) and secondarily exercise fidelity (i.e., proportion of prescribed exercise repetitions completed with high quality based on predefined movement acceptability criteria) when using Bootle Boot Camp with and without movement-tracking feedback.
2. Compare children’s affective and cognitive engagement (i.e., smiley face ratings and survey results) when using Bootle Boot Camp with and without movement-tracking feedback.

Qualitative objective was to:

3. Explore children’s and parents’ home exercise experiences when using Bootle Boot Camp with and without movement-tracking feedback.

It was hypothesized that behavioural, affective and cognitive engagement would be higher when children played Bootle Boot Camp with the movement-tracking feedback enabled.

## Methods

The full study trial protocol has been previously reported [21]. This paper reports the results of three child-parent dyads participating in the Bootle Boot Camp trial. Reporting follows the National Institute of Health 2011 document outlining best practices for health sciences mixed methods research [22], Good Reporting of a Mixed Methods Study (GRAMMS) [23], and the Single-Case reporting Guideline in Behavioural Interventions (SCRIBE) [24] and Consolidated Criteria for Reporting Qualitative Research (COREQ) [25] to guide reporting of quantitative and qualitative strands.

### Research design

A multi-case mixed methods study design using an explanatory sequential approach was used, whereby quantitative data collection and analysis were completed first, followed by qualitative data collection and analysis to help explain and elaborate on quantitative findings [26–28]. This design was selected to enable an in-depth understanding of children’s and parents’ complex experiences with Bootle Boot Camp using multiple types of data across cases [28]. Integration, as guided by a pragmatic research paradigm [29], occurred during study planning with a mixed methods study design and research objective, and at the methods level where a semi-structured qualitative interview guide was *built* from the quantitative data [28, 30]. During the interpretation and reporting phase, narrative weaving (quantitative and qualitative results reported on a concept-by-concept basis) and joint displays were used to *merge* quantitative and qualitative data to gain new rich insights (i.e., metainferences) and determine the extent to which data types confirmed, contradicted or expanded understanding [28, 30–33]. 

### Intervention

Bootle Boot Camp is an ICP-HEP that was created using the five stages of design thinking (empathize, define, ideate, prototype, and test) [34, [35], and in consultation with clinician and family knowledge holder partners, game designers, digital artists, researchers and engineers at Holland Bloorview Kids Rehabilitation Hospital (Holland Bloorview) and in consideration of self-determination [36] and motor learning theories [37]. A full description of Bootle Boot Camp, its design process, feedback elements, and game features is provided elsewhere [18], with a brief summary offered below.

Bootle Boot Camp enables clinicians to prescribe individualized HEPs to clients in a gamified format using an online web-interface application, with lower limb range of motion, strengthening, balance, cardiorespiratory fitness and flexibility exercises available. Clinicians select the exercises to include within the HEP, the number of repetitions and sets or timed durations (e.g., stretches), and whether the exercise should be performed supported (holding on to a chair/walker) or unsupported. Children are guided on appropriate exercise performance through exercise videos alongside a live video feed of their own movements, enhanced with a skeletal joint overlay. General exercise instructions are also available throughout practice and can be accessed by clicking on the ‘Help’ button. Interactivity, choice and social play are promoted through the child’s selection of a robot avatar to exercise with, game mode to play (e.g., "Guess the Bootle," "Would You Rather," and "Fact or Fiction") and player mode (single vs. multiplayer), with both players performing the same set of exercises. Short- and long-term rewards are designed to improve motivation, with badges awarded for the completion of all exercise sessions in a single week, and streaks awarded for continuous session completion across weeks. Bootle Bucks are also provided for exercise completion, with more Bootle Bucks awarded for exercises that are performed with high quality in the feedback version of the game. These can be used to purchase items from the Bootle Bootique, including different backgrounds and music to exercise to, as well as different pets and accessories for the robot. Finally, the game aims to optimize safety through the presence of a speed indicator that detects increased speeds and issues a warning to the player to slow down to minimize falls risk and potential injury. Rest breaks are also offered after each exercise, where the child must raise their hand to continue game play.

The game is played on a television with a 3D camera-computer system, the Orbbec Persee+ (https://orbbec3d.com). The Orbbec Persee + integrates an Astro Pro 3D camera with a built in ARM processor and graphical processing unit. The Orbbec Body Tracking software development kit is used to identify the x, y, z coordinates (i.e., horizontal, vertical and depth positions) of 19 joints of the participant’s body at a rate of 30 frames per second [19, 38]. Joint positions, joint angles, and movement speed are used to determine successful movement execution in the game based on predefined movement acceptability criteria developed by authors MP and FVW and in consultation with five external PTs. In the feedback version of the game, feedback is provided based on these criteria to further enhance movement quality and safety. This movement-tracking feedback is delivered through prescriptive visual/audio cues provided by virtual Coach Botley related to movement execution and quality (e.g., ‘bend your hips and knees’ and ‘keep your body straight’), visual indicators (e.g., degree of truncal lean), repetition counters that count the number of high-quality repetitions, and a 3-star rating system reflecting movement quality (i.e., 1 star awarded when less than 50% of repetitions are performed with appropriate form, 2 stars for 50–75%, and 3 stars when more than 75% of repetitions are performed with appropriate form). Awarding 1 star, even in the event of suboptimal performance, aims to motivate a child and foster continued participation and engagement with the exercise program. There is also a self-selected optional exercise summary screen at the end of the session outlining movement metrics performed well and those requiring improvement, with star ratings across exercises and sessions displayed graphically.

### Procedures

#### Patient and public involvement statement

Knowledge holders were involved in study design and advised on the relevance of the research, priority of research question/objectives (e.g., emphasized importance of adherence primarily, followed by exercise fidelity), eligibility criteria, recruitment strategies (e.g., dual recruitment pathways for families and clinicians to improve study access), and study plan feasibility (e.g., confirmed four weekly exercise sessions would be manageable if HEPs were limited to 30 min per session). Specifically, a pediatric PT, 12-year old child with CP, and his mother served as knowledge holders, as guided by the Ontario Brain Institute’s framework for community member participation in research [39] and the family engagement in research resource developed by CanChild [40]. Gift cards were issued to knowledge holders as a form of remuneration for their study involvement. The child and his parent took part in knowledge translation activities, with both participating in a multidisciplinary game development panel as part of an engineering course at the University of Toronto, and the parent additionally providing insights into the family experience with game development as part of an interdisciplinary research panel at a local conference. Following study completion, these knowledge holders were given opportunities to provide feedback and contribute their perspectives to our data interpretation, however they opted out of this next stage.

## Phase 1—quantitative

### Design

A semi-randomised, non-blinded, single-case experimental design (SCED) with alternating-treatments (ATD) [41, 42] was used to primarily determine the impact of feedback on exercise adherence, a reversible behaviour. The comparison period consisted of Bootle Boot Camp exercise sessions with movement-tracking feedback alternated over a four-week period with Bootle Boot Camp sessions without movement-tracking feedback. Restricted randomized treatment schedules (limit of two maximum consecutive administrations of the same treatment condition) were predetermined for each participant using R software. This randomization process was used to avoid long sequences of consecutive administration of the same condition and minimize threats to internal validity (e.g., history, maturation, order effects) [42–45]. Participants were aware of the treatment condition by the presence or absence of virtual Coach Botley, who on feedback provision days was present to count the number of exercise repetition attempts and high-quality repetitions performed by the child that met the movement criteria (exercise fidelity) built into the system, and provide corrective feedback and encouragement.

The superior intervention (movement-tracking feedback or no feedback), as determined for each child (explained in methods), was then offered for two additional weeks (i.e., referred to as ‘best alone’ phase) to detect potential multitreatment interference during the comparison phase, such as carryover effects (i.e., the effect of one intervention condition on performance during another intervention condition) or rapid alternation effects (i.e., the effect on performance due to rapid changes in intervention conditions) [42, 46]. If engagement data remained similar during the comparison and best alone phases, multitreatment interference was unlikely to have occurred [46]. The child and family’s understanding of the presence/absence of feedback was confirmed by the student investigator/first author (MP) during the family’s home onboarding session.

### Recruitment

Child-parent dyads were recruited through community communication channels (e.g., Bloorview Research Institute ‘Participate in Research’ webpage, CP-Net newsletter) and through physiotherapy-related community channels (e.g., Holland Bloorview, Canadian Physiotherapy Associated Pediatric Division, OT/PT Pediatric Network). The eligibility criteria were expanded from the published protocol [21] after interest was expressed from non-eligible participants during the third month of enrollment. Children ages 6–14 years (beyond initial age limit of 12 years), with a diagnosis of CP or non-progressive neuromotor disorder (e.g., spina bifida, acquired brain injury, pediatric stroke), able to ambulate safely and independently on level surfaces without the use of a handheld mobility device (e.g., GMFCS levels I-II [20] or similar functional level as determined by the child’s treating physiotherapist) if external to Holland Bloorview, or GMFCS levels I-III if an internal client, and on or off active physiotherapy were eligible. Additionally, children had to have at least one goal related to the lower limb, be able and willing to engage in four weekly exercise sessions for six weeks, speak and understand English, have a parent able to participate, and have access to home technology and internet services for phone or video conference calls to be eligible. Eligibility was limited for the children in GMFCS level III to be those who were located within Holland Bloorview’s catchment area to enhance safety as these children are at higher risk for falls compared to those in levels I and II and the research team’s ability to respond to adverse events in a timely fashion given this was the first time trialing this technology in participants’ homes. The child’s treating PT had to also be willing to participate to prescribe an individualized HEP using Bootle Boot Camp tailored to the child’s needs. Exclusion criteria included: botulinum neurotoxin type A (BoNTA) injection in the previous 12 weeks or orthopedic surgery in the previous 6 months, serial casting, orthopedic surgery, serious medical intervention or extended event (e.g., family trip) during the intervention period, photosensitivity or unstable epilepsy triggered by screen activities, visual or auditory deficits that would interfere with game play, respiratory, cardiovascular or other medical condition that might make gameplay unsafe, or intensive intervention schedule (i.e., more than 3 times per week). MP confirmed child and parent interest in participating, provided study information and obtained consent/assent via Zoom videoconference for those who wished to proceed.

### Sample size

In quantitative SCED research, power is derived from the number of repeated measurements of a target behaviour, rather the number of participants [47, 48]. SCED standards recommend at least three demonstrations of effect at three different time points to establish causal relation [49]. Three to four child participants per gender and age stratum (6–8 years) and (9–13 years) were initially sought, as age and gender have been shown to influence physical activity levels and time spent playing virtual reality games [50–52]. However, further recruitment was stopped after three children were enrolled to enable technology refinements based on these users’ experiences.

### Intervention and data collection

#### In-person session with PT

PTs were given a Bootle Boot Camp training manual and operational video to familiarize themselves with the exercise prescription web interface. The PT scheduled one in-person session with their client to establish three lower limb goals, set up an individualized HEP using Bootle Boot Camp based on their needs and abilities (i.e., exercises, the limb(s) to be targeted, repetitions, sets, need for hand hold support to be completed) four times per week for six weeks, and provide education on the smiley face rating scales used throughout the study. PTs were instructed to prescribe HEPs that could be completed within 30 min, based on knowledge holder input. Detailed exercise plans are available in the supplementary material (Online Appendix 1). MP attended this session virtually and answered clinician and family questions.

#### Home onboarding

Participating families were mailed a Bootle Boot Camp kit (i.e., Orbbec Persee + and Bootle Boot Camp Family User Guide). The child and parent were then contacted by MP to schedule a virtual one-hour home onboarding and exercise session with members of the research team (MP and AK). During the onboarding session, the family was guided through technical set up (e.g., camera positioning) and instructed on how to prepare the environment (e.g., remove clutter, avoid low light) and child (e.g., wear tight-fitting clothing) for exercise. During this time, the research team also set expectations (e.g., therapy focus of game rather than entertainment), provided general instructions on system use (e.g., exit game fully before turning off system), and responded to questions. The child then completed their first exercise session, with MP and AK explaining game features as encountered.

#### Comparison phase (weeks 1–4)

For the first four weeks of training, each child completed their individualized HEP using Bootle Boot Camp with and without movement-tracking feedback (same exercises each session), with the two game versions alternating across sessions based on each child’s pre-defined randomization schedule. Weekly email reminders were sent to parents to complete exercise sessions. During exercise sessions, children’s body joint data and exercise performance were tracked and video recorded by the Orbbec Persee+. Since Bootle Boot Camp was designed with cloud functionality, researchers could remotely track game usage on a weekly basis and update game settings as needed. On feedback days, body joint data were used to provide feedback on exercise performance and exercise results during gameplay (e.g., truncal lean indicator, repetition counter recording number of quality repetitions completed) while on no-feedback days, these data were still collected but not reported to the child. In the feedback condition, children were encouraged to trial exercise repetitions through a 10-second delay in the appearance of the ‘Next exercise’ button. If this was not used, the child was allowed to complete three extra repetition attempts beyond what was prescribed to try and perform movements with optimal quality before the game would progress to the next prescribed exercise. For timed exercises (e.g., stretches), children were given three attempts over a 2-minute period to achieve their best exercise performance, in alignment with the *Challenge* [53], a measure of advanced gross motor skills in children with CP, before the game advanced to the next exercise. Setting these repetition and time caps was meant to ensure that each child did not spend too long on any one exercise to avoid frustration or potential injury from excessive practice. Clinicians were alerted to these restrictions when prescribing HEPs. In the no-feedback condition, the child counted their own repetitions and self-reported exercise completion by pressing the ‘Next Exercise’ button to advance to the next exercise, with time-based exercises counting down automatically.

#### Best alone phase (weeks 5–6)

At the end of week 4, MP reviewed the child’s exercise repetition attempts data to determine which version of the game resulted in the highest exercise adherence. The percentage of non-overlapping data (PND) was calculated, with 90% of non-overlap between adherence data paths considered indicative of the ‘superior’ intervention [46, 54]. If this was not met, the highest mean proportion of prescribed exercise repetitions attempted was used to determine the game version that would be carried out for the following two weeks. The best-alone version was configured for the child, with AK updating the game settings remotely. The family was notified of this decision via email. At the start of the following week, parents were sent a secure study-specific acceptability survey for their children to complete via Research Electronic Data Capture (REDCap) [55, 56] tools hosted at Holland Bloorview.

### Questionnaires and outcome measures

#### Demographics

Demographic data were collected using REDCap tools. Child measures, including the Pediatric Evaluation Disability Inventory Adaptive Test (PEDI-CAT) ( rather than speedy version ) [57], the revised Physical Activity Enjoyment Scale (PACES) [58], and the Children’s Self Perceptions of Adequacy in and Predilection for Physical Activity Scale (CSAPPA) [59] were also completed online at this point by the children with support from their parent to provide insights into their baseline level of function, physical activity enjoyment and self-efficacy, respectively.

#### Measures of engagement

Behavioural engagement (objective 1):

*Exercise adherence*

Exercise adherence was expressed as a proportion based on the number of exercise repetitions attempted or duration of attempts (for timed exercises) divided by the number/time prescribed for each exercise session. A movement was counted as a repetition *attempt* if it met all predefined attempted repetition metrics for each exercise by the group of experienced PTs (e.g., for a dynamic exercise such as the squat, a minimum of 20 degrees of hip and knee flexion with the head/chest displacing to a lower position than at starting). For a static (timed) exercise, such as tandem stance, a child had to step forward or backward with one leg/foot to achieve a staggered stance) (Table 1) [21]. If a child attempted more repetitions than what was prescribed, this value could be greater than 1.0.

**Table 1:** Weighted mean relative error (WMRE) comparing manual and system attempted repetition counts for each exercise

Exercise	Attempted exercise repetition metric(s)	Child 01 WMRE (%)	Child 02 WMRE (%)	Child 03 WMRE (%)
Hip flexion (bilateral)	☐ Lifts one leg to achieve at least 20 degrees of hip flexion ☐ Lifts other leg to achieve at least 20 degrees of hip flexion	21.1		
Hip abduction	☐ Lifts one leg out to the side to achieve at least 20 degrees hip abduction	14.2		
Forward step	☐ Steps forward with one leg/foot to result in staggered stance (toes of one foot at or beyond toes of other foot)		10.7	27.5
Lateral step	☐ Steps laterally with at least one leg/foot (foot beyond shoulder)	89.5		38.0
Backwards step	☐ Steps back with at least one foot (minimum one foot length distance achieved)	23.0	29.0	
Tandem stance	☐ Steps forward or backward with one leg/foot to achieve staggered stance (heel of one foot at or beyond toes of other foot)			57.1
Single leg stance	☐ One-foot lifts off the ground (single limb stance detected)	47.4		
Sit to stand	☐ Head/chest displace vertically (to a lower position during sit and to a higher position during stand)		19.4	2.5
Calf stretch	☐ Steps back such that one leg is behind the other (staggered stance)		11.8	
Hamstring stretch	☐ Chest displaces anteriorly while child is seated on the floor		100.0	
Squat	☐ Head/chest displace vertically to a lower position ☐ At least 20 degrees of hip and knee flexion achieved	26.4		
Seated star jump	☐ At least one arm and foot move laterally (away from trunk)			26.0
Kick	☐ Brings one leg anteriorly (foot in front of the body)	74.4		19.2

Manual repetition counts by rater MP were treated as ground truth compared to counts by the Orbbec Persee + system. Metrics used to quantify an exercise repetition attempt are shown

*Exercise fidelity*

Exercise fidelity was expressed as a proportion based on the number of high-quality repetitions (movement fidelity) divided by the number of repetitions prescribed for each exercise session. An exercise repetition was considered *high-quality* if it met a set of quality repetition metrics predefined for each exercise by the group of experienced PTs. For example, for the squat exercise, these criteria required that the child’s feet be shoulder width apart and unmoving, knees not touching and in line with ankles, head/chest displace vertically, hips/knees achieve 90 degrees flexion in lowest position within a 20-degree range and less than 10 degrees lateral truncal lean. For timed exercises, quality was defined as the best time achieved over three attempts divided by the time prescribed. For example, for the tandem stance exercise, criteria required that the child’s front leg achieve a minimum of 15 degrees hip flexion and 5 degrees hip adduction (used as a proxy for the heel of the front foot exceeding the toes of the hindfoot), with the position held for the duration set by the child’s physiotherapist. For more examples of quality metrics used to determine exercise fidelity, readers are referred to previous work on this [19]. If a child performed more high-quality repetitions that what was prescribed, this value could be greater than 1.0.

Affective and cognitive engagement (objective 2):

*Fun and helpfulness ratings*

Following each exercise session, children rated their perceived level of fun (affective engagement) and helpfulness for the body (cognitive engagement) directly in the game using two different 5-point smiley face rating scales based on the Smileyometer [60, 61]. Scales were customized to each child during the onboarding session with the PT, where the child was asked to select the most fun and least fun activities and most helpful and least helpful activities for their bodies, in line with the Personalized Enjoyment Questionnaire [62], to improve scale understanding and responsiveness. These data were supplemented by the Bootle Boot Camp Acceptability Survey that was completed by children via REDCap at the end of the four-week comparison period. The survey consisted of 5-point ordinal rating scales (1-disagree, 2-somewhat disagree, 3-neither agree nor disagree, 4-somewhat agree, 5-agree) for 19 items, open-ended response sections, and selection of the preferred game version (movement-tracking feedback or no feedback).

### Data analysis

#### Visual analysis

The proportion of attempted exercise repetitions (exercise adherence), and smiley-face ratings (affective/cognitive engagement) were plotted across exercise sessions, with graphs inspected visually for level (low, moderate or high), trend (direction and magnitude), variability (highly variable, somewhat variable, stable) and overlap (PND) [43, 46, 49]. Since exercise fidelity may be considered a non-reversible behaviour (e.g. learnings from the feedback condition may carry over to the no-feedback condition), statistical analysis was used in place of this visual analysis approach.

#### Statistical analysis

Descriptive statistics were used to summarize exercise attempts or timed durations, high-quality exercise attempts, smiley-face ratings, and survey data for each participant in the feedback and no- feedback treatment conditions.

##### Objective 1a (adherence)

For exercise adherence data (interval), nonparametric one-tailed single case randomization tests (SCRT) were conducted to determine if the mean proportion of prescribed exercise repetitions attempted in the feedback condition was higher than in the no-feedback condition, beyond what would be expected by chance [44, 63]. In cases with a small sample size (i.e., fewer than five feedback or no-feedback exercise sessions for a child), the Bayesian t-test was used to compare this difference, with the Bayes factor (BF) quantifying the evidence for or against the null hypothesis [64, 65]. 

##### Objective 1b (exercise fidelity)

To compare the difference in overall movement quality between treatment conditions, session-level mean proportions of quality repetitions across all exercises were compared using a nonparametric one-tailed approximate Fisher-Pitman permutation test (Fisher-Pitman test) in cases with unpaired data (i.e., unequal number of feedback and no-feedback exercises sessions) [66]. In cases with paired data (i.e., equal number of feedback and no-feedback sessions), a nonparametric one-tailed approximate Wilcoxon-Pratt signed-rank test (Wilcoxon-Pratt test) was used [67], with a significance level of 0.05 applied for all tests. This was necessary in case a child did not complete all exercise sessions as intended, resulting in unbalanced feedback and no-feedback sessions for comparison.

##### Objective 2 (perceived fun and helpfulness)

For the fun and helpfulness data (ordinal), a one-tailed Fisher-Pitman test was used to compare median differences in cases with unpaired data, and a one-tailed Wilcoxon-Pratt test in cases with paired data.

#### Deviations from protocol

The published protocol [21] described that a battery of clinical tests (i.e., the Five Time Sit to Stand Test [68], the modified Timed Up and Go [69, 70], the One Leg Stance Test [71], the Pediatric Reach Test [72] and the 30 Second Sit to Stand Test) [73, 74] would be administered during exercise sessions in weeks 1 and 6 to evaluate lower limb treatment response as part of quantitative objectives, with goal achievement measured using the Canadian Occupational Performance Measure (COPM) [75–77]. These clinical measures, including COPM scores, will be reported in a parallel paper along with the lessons learned from administering virtual assessments in this way. However, families’ qualitative reflections on goal progress and value of the intervention are included in this report, where available.

#### Post hoc analysis

Weekly review by MP of exercise videos captured by the Bootle Boot Camp system revealed potential limitations in movement tracking accuracy due to varying play environments (e.g., low light conditions, children wearing baggy clothing, pets and toys in play space). In some cases where repetition counts were not being registered, parents were observed to trial exercises to solve the issue, with the system not distinguishing between the child’s attempts and the parent’s attempts. Since this, in combination with poorly tracked exercises, had the potential to impact system-tracked exercise adherence and fidelity results, manual counts of exercise repetitions were implemented following completion of the third child’s study participation to assess the level of agreement between manual and system counts. MP reviewed each child’s exercise videos twice, manually counting/timing exercise repetition attempts and quality repetitions/times. Agreement of manually counted repetitions and timed exercises between MP and the automated exercise counts of the Orbbec Persee + was assessed using weighted mean relative error (WMRE) across child participants [78]. WMRE measures the weighted mean of the relative errors using manual counts as ground truth. While there is no established acceptability threshold for repetition errors and children are expected to vary widely in their tolerance for system errors, for the purpose of this work, we considered a WMRE of less than 25% to be acceptable. A greater error would lead to inaccurate movement quality results and feedback being presented to the child, i.e., with the system’s three-star movement quality ratings, if the exercise repetition attempts were off by more than this 25% threshold, a child would automatically be put in a different movement quality category regardless of their actual performance, leading to inappropriate feedback being triggered/given.

To evaluate and hopefully confirm the consistency of MP’s manual ratings to permit their subsequent use, inter-rater reliability between MP and an external PT (PA) who has experience working with children with CP and rating movement quality, was evaluated. A one-hour training session was held with MP, EB, FVW and PA where a sampling of 15 child participant exercise videos was reviewed alongside movement acceptability criteria, and the scoring process explained and practiced. Thirty videos across the child participants during their six weeks of exercise practice and different from those used for training were then randomly selected by AK, with thirty videos adequately powering this reliability study [79]. The first 10 exercise repetitions attempted and first 10 s of timed exercises were scored by MP and PA independently (to ensure that each scored the same video segments of these participants who were prescribed different exercise parameters), with MP and PA blinded to treatment conditions and session number. Each video was immediately reviewed a second time by raters to count/time quality repetitions. An intraclass correlation coefficient (ICC) (2,1) was used to enable generalizability of findings to other PT raters [80]. ICC estimates and their 95% confidence intervals were calculated using R software and Bland-Altman plots constructed to estimate rater agreement, with an ICC of 0.90 or greater indicative of excellent reliability and 0.75 to 0.90 signifying good reliability [80, 81]. 

### Safety monitoring and adverse events

A safety monitoring committee consisting of a PT, pediatrician and researcher, was established to review any potential adverse events during home training. Details of this process are available in the published protocol [21]. 

### Integration point: building of the interview guide

Engagement and survey data were used to build the qualitative interview guide to help contextualize and understand engagement outcomes more robustly (e.g., ‘You indicated in the survey that the no-feedback version was more fun to play. Can you tell me why it was more fun to play?’) (Online Appendix 2).

## Phase 2—qualitative

### Design

A qualitative descriptive design was employed [82, 83], with families interviewed to better understand their experiences with the two versions of Bootle Boot Camp.

### Participants and sample size

Purposive sampling was used where all children and parents were invited to take part in interviews in phase 2 of the study. Child 02 opted to not take part in the interview, which was conducted individually with her parent. All other interviews were dyadic.

### Data collection

#### Follow up period (weeks 7 and 8)

Following six weeks of training, families returned the Bootle Boot Camp kits. The child and parent were then contacted by MP to schedule their interview. Semi-structured interviews were conducted with children and their parents over Zoom videoconferencing by MP, with children given the option of individual or dyadic interviews. Dyadic interviews have been shown to support rich data collection with young children, with parents helping to enhance stories and trigger important memories [84, 85]. A combined individual and dyadic interview approach has been used in a previous study exploring children with CP’s engagement with a home-based ICP technology [12]. Interviews were 60–90 min in duration and were audio recorded.

### Data analysis

Interview data were analysed using directed content analysis [86]. Audio recordings of interviews were transcribed verbatim by MP, with members of the analysis team (MP, IF, SS, EB) reading interviews in full. Two independent coders (MP and IF) coded the first interview using NVivo 12.0 software using a preliminary codebook developed deductively using King’s engagement framework [10], with additional codes identified inductively. Coders meet to discuss codes and update the codebook based on consensus. MP and SS then coded all remaining interviews, meeting after each one to discuss coding decisions and update the codebook. Codes were collated into subcategories by MP, IF and SS before being mapped to engagement categories by MP. The team met to discuss codes, subcategories and categories before a final list was agreed upon. Rigor was enhanced through implementation of NVivo 12.0 computer software, an audit trail, triangulation and negative case analysis [87]. A sample of the codebook is presented in Online Appendix 3.

#### Positionality

MP is a female registered PT with clinical experience in the assessment and treatment of children with neuromotor disorders. MP is also a research trainee with some qualitative interviewing and mixed methods research experience. MP conducted all interviews and was involved in all levels of interview analysis. MP’s clinical experience, involvement in all stages of study design and implementation, and pragmatic research approach may have impacted the probes used during interviews and the insights shared by families based on their pre-existing relationship with MP. These potential biases were mitigated by having a multidisciplinary analysis team that included researchers with nonclinical backgrounds (SS and EB, rehabilitation engineering) and clinical backgrounds (IF, kinesiology, and FVW, physiotherapy) involved in the development of the interview guide and analyses.

### Integration point: narrative weaving and use of joint display to describe experiences

Quantitative engagement and qualitative interview data were integrated through narrative weaving and the use of joint displays, with metainferences generated and classified as confirmed (findings from quantitative and qualitative data are in agreement), discordant (findings from data sources conflict) or expanded (findings expand understanding) [28, 30–33]. 

## Results

### Recruitment

Three child-parent dyads participated as described in Table 2, with four other children who had expressed interest in participating considered not eligible (1 outside of Canada, 1 functional level and 2 diagnosis). One other child-parent dyad declined participation as they were unable to commit to the six-week study duration at the time of recruitment.

**Table 2:** Child-caregiver dyad demographics

Child participants												
ID	Age (years)	Gender	Diagnosis	GMFCS Level	Revised PACES^a^	CSAPPA^b^	Speedy PEDI-CAT^c^				Child-reported weekly video game use	Child-reported weekly home exercise time (pre study)
							Domain		T Score	Percentile		
C01	12	Boy	Cerebral palsy: bilateral, mixed type	II	2.6	Adequacy: 11 Predilection: 16 Enjoyment: 7 Total Score: 34	Daily activities		< 10	< 5	7 h	0.33 h
							Mobility		< 10	< 5		
							Social / cognitive		32	< 5		
							Responsibility		37	5 ~ 25		
C02	13	Girl	Cerebral palsy: spastic quadriplegia	II	–	–	–				18 h	1 h
C03	9	Girl	Acquired brain injury: left sided hemiplegia	II	2.9	Adequacy: 7 Predilection: 14 Enjoyment: 3 Total Score: 24	Daily activities		13	< 1	4.5 h	0 h
							Mobility		< 10	< 1		
							Social / cognitive		24	1		
							Responsibility		34	4		

Parent participants												
ID	Age range (years)	Gender	Relationship to child	Ethnicity	Annual household income		Level of education	Work status	Family unit		Comfort with using new technology (1 = not comfortable, 5 = very comfortable)	
P01	41–50	Woman	Mother	Asian	$50,000–$74,999		Bachelor’s degree	Self-employed	2 Adults, 2 children		3	
P02	41–50	Woman	Mother	Caucasian	$100,000–$149,999		College certificate	Full time employee	2 Adults, 2 children (1 young adult)		4	
P03	31–40	Woman	Mother	Middle Eastern	$100,000–$149,999		Bachelor’s degree	Full time employee	2 Adults, 2 children		4	

GMFCS: Gross Motor Function Classification System, PACES: Physical Activity Enjoyment Scale, CSAPPA: Children’s Self-Perceptions of Adequacy in and Predilection for Physical Activity, Speedy PEDI-CAT: Speedy Pediatric Evaluation of Disability Inventory - Computer Adaptive Test, HEP: home exercise program

^a^Revised PACES: average scoring of 16-items rated from 1 = totally disagree to 5 = totally agree

^b^CSAPPA: scores range from 19–76, with ≥ 60 indicative of high self-efficacy

^c^Normative standard scores (T-scores and age percentiles) describe a child’s performance relative to other children of the same age. T-scores between 30–70 (mean+/-2 SD) are within expected range, while scores below 30 are suggestive of reduced functional ability. Age percentiles represent the percentage of children of the same age bracket whose scores were as high or higher than the target participant, with scores below the 5th percentile considered below average

– Indicates measure not completed by child/family

Two of the three participating dyads took part in full (01 and 03), while one dyad (02) opted to not complete all demographic measures, post-comparison period surveys or the post-intervention interview (child 02).

### Reliability

#### Agreement (between manual and system counts)

Variability in movement tracking reliability was observed across exercises and child participants. While exercises such as sit to stand, forward step and calf stretch tracked reliably (maximum WMRE = 2.5%, 10.7% and 11.8% respectively), others demonstrated poor tracking for some participants: lateral stepping and kicking (child 01: WMRE 89.5% and 74.4%), hamstring stretch (child 02: WMRE 100%), and tandem stance (child 03: WMRE 57.1%) (Table 1). Given that some exercises exceeded our acceptable error threshold of 25%, the manual exercise counts of MP were used to determine exercise adherence and fidelity for all child participants and exercises.

#### Inter-rater reliability (between PT raters)

The ICC (2,1) for attempted repetitions across exercises was 0.86 (95% CI [0.74, 0.93], *p* < 0.001) and 0.93 for quality repetitions (95% CI [0.86, 0.97], *p* < 0.001), indicating good and excellent reliability between raters, respectively. The Bland-Altman plot for attempted exercise repetitions did not show any scoring bias (Online Appendix 4), while the plot for movement quality showed some potential scoring bias when fewer quality repetitions were recorded (Online Appendix 5).

### Facets of engagement and participant experiences

Given the multi-case mixed methods explanatory sequential study design, quantitative and qualitative findings have been integrated and presented for each participating dyad on a construct-by-construct basis through narrative weaving and joint displays.

## Child-parent dyad 01

Child 01 set three lower limb goals with his PT: (1) to be able to run faster, (2) to walk more than 1 km with friends, and (3) to trip less when walking/playing on grass. His PT prescribed 2 sets of 6 exercises (i.e., hip flexion, lateral step, hip abduction, squat, kicking, backwards stepping) and 3 sets of 1 exercise (i.e., single leg stance) for each session. During week 2, the family requested to have the HEP shortened to a 1-set plan, in consultation with the child’s PT. The full plan was then reinstated during week 4 at the request of the parent and child after sessions were reportedly too short.

This child completed 13 of the 16 prescribed exercise sessions during the comparison phase and completed 11 smiley face session ratings. He had a mean active exercise play time of 7.36 min (SD 2.34 min) in feedback sessions and 8.77 min (SD 4.19 min) in no-feedback sessions. Technical issues reported by the family via email (i.e., game freezes) during weeks 2 and 4 prevented completion of 3 sessions. Based on the mean proportion of prescribed exercise repetitions attempted, the feedback version was used for the best-alone phase. During this 2-week phase, the child completed 6 of 8 possible sessions with a mean active play time of 9.25 min (SD 1.73 min). Technical issues in week 6 (i.e., no game sound, frozen screen), prevented completion of two sessions, with movement tracking issues limiting repetition attempts during one other session (session 21).

### Behavioural engagement

#### Exercise adherence

##### Quantitative

**Fig. 1:**
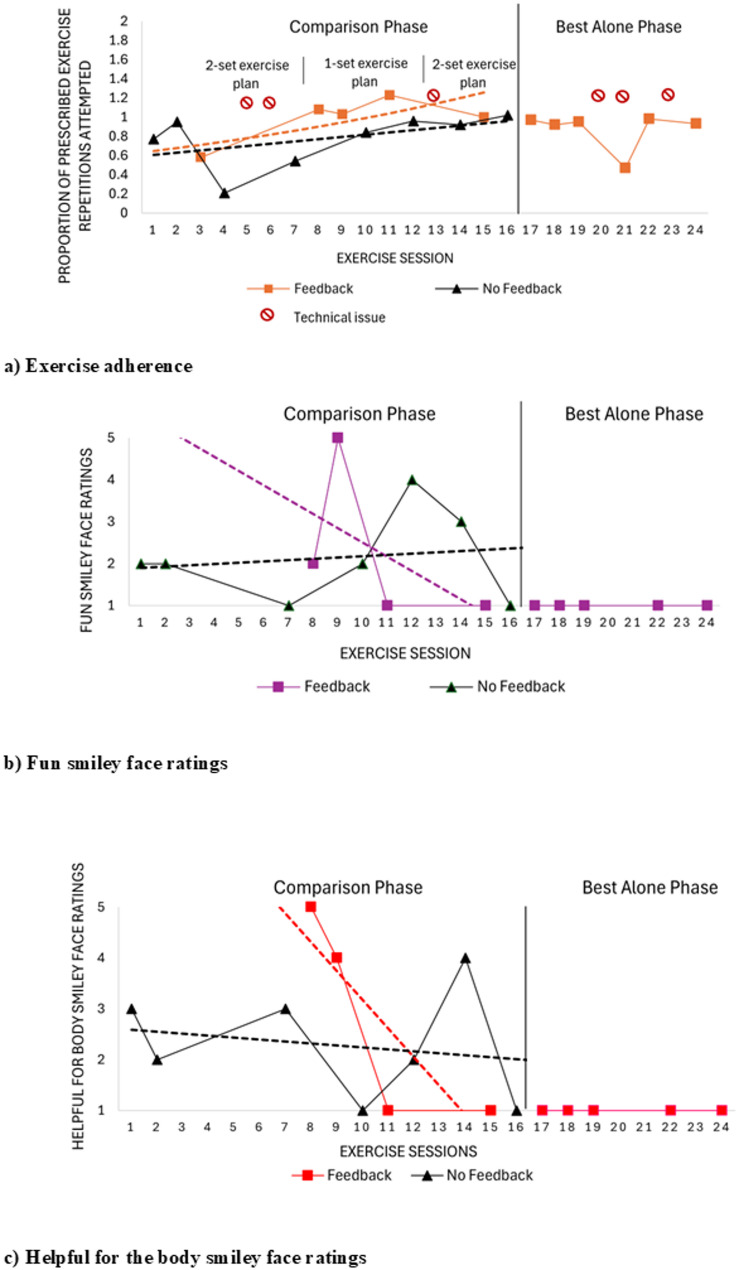
Engagement outcomes for child 01. **a** Exercise adherence (i.e. proportion of prescribed exercise repetitions attempted) as a measure of behavioural engagement, where a value of 1.0 indicates completion of all prescribed exercise repetitions and sets across exercises and a value greater than 1.0 indications completion of more exercise repetitions than what was prescribed. **b** Fun smiley face ratings (i.e. affective engagement) across exercise sessions where 1 = no fun at all and 5 = lots of fun. **c** Helpfulness for the body smiley face ratings (i.e. cognitive engagement) across exercise sessions where 1 = not helpful for the body and 5 = very helpful for the body

Levels of exercise adherence were higher and accelerating in the feedback condition relative to the no-feedback condition, with this differentiation generally consistent across sessions (Fig. 1a; Table 3). The PND was 80%, indicating that adherence was higher in the feedback condition for 4 out of 5 between-session comparisons (i.e., 4 demonstrations of functional relation). The child achieved a mean exercise adherence (i.e., proportion of prescribed exercise repetitions attempted) of 0.98 (SD 0.24) in the feedback condition and 0.77 (SD 0.27) in the no-feedback condition, with a SCRT demonstrating a significant difference between conditions (*p* < 0.001; Table 3), unlikely to have occurred due to chance. Mean adherence was 0.87 (SD 0.19) during the feedback-only best-alone phase.

**Table 3:** Visual/statistical analysis of exercise adherence and statistical analysis of exercise fidelity across child participants

	Child 01			Child 02			Child 03		
	Comparison (4 weeks)		Best alone (2 weeks) F	Comparison (4 weeks)		Best alone (2 weeks) NF	Comparison (4 weeks)		Best alone (2 weeks) F
	F	NF		F	NF		F	NF	
*Exercise adherence* ^a^									
Sessions (n)	5	8	6	4	2	4	6	6	3*
Level	High	High	High	High	High	High	High	High	High
Trend	Gradual accelerating	Gradual accelerating	Zero celerating	Zero celerating	Gradual accelerating	Gradual decelerating	Zero celerating	Gradual decelerating	Gradual decelerating
Variability	Somewhat variable	Somewhat variable	Stable	Stable	Stable	Stable	Somewhat variable	Highly variable	Stable
PND (%)	80.00		n/a	0.00		n/a	83.30		n/a
Mean	0.98	0.77	0.87	0.82	0.86	0.78	1.21	0.94	1.21
SD	0.24	0.27	0.19	0.09	0.15	0.15	0.30	0.80	0.18
Median	1.03	0.88	0.94	0.84	0.86	0.77	1.15	0.84	1.23
Min / Max	0.58 / 1.23	0.21 / 0.96	0.47 / 0.97	0.70 / 0.90	0.76 / 0.97	0.60 / 0.96	0.93 / 1.21	0.27 / 2.47	1.02 / 1.39
p-value^+^	< 0.001		n/a	BF = 0.62		n/a	0.57		n/a
*Exercise fidelity* ^b^									
Mean	0.34	0.30	0.39	0.34	0.30	0.36	0.71	0.35	0.71
SD	0.08	0.09	0.04	0.16	0.08	0.06	0.14	0.08	0.13
Median	0.35	0.30	0.40	0.33	0.30	0.38	0.70	0.35	0.69
Min / Max	0.24 / 0.44	0.16 / 0.39	0.34 / 0.46	0.18 / 0.54	0.24 / 0.36	0.28 / 0.41	0.53 / 0.89	0.22 / 0.45	0.59 / 0.85
p-value^++^	0.19		n/a	0.46		n/a	1.0		n/a

F = feedback, N = no feedback

^a^Exercise adherence is described as the proportion of prescribed exercise repetitions attempted

^b^Exercise fidelity is described as the proportion of prescribed exercise repetitions completed with acceptable form based on predefined acceptability criteria

*Technical glitch resulted in 1 no-feedback exercise session being provided in the feedback-only best alone phase. Measures of central tendency shown are in consideration of the 3 remaining feedback sessions in this phase

^+^Single-case randomization test (SCRT) was used to determine if the mean difference in exercise adherence was statistically significant, with a level of significance of 0.05. For child 02, a Bayesian t-test was used in place of the SCRT due the small sample size, withthe Bayes Factor (BF) shown

^++^ Approximate two-sample Fisher-Pitman permutation test was used to assess if the mean difference in exercise fidelity was statistically significant in cases with unpaired data (unequal number of feedback and no feedback sessions) (i.e., child 01 and 02), with the Approximate Wilcoxon-Pratt Signed-rank test used to assess this difference for paired data (i.e., child 03). A level of significance of 0.05 was applied

##### Qualitative

The child expressed that he primarily played Bootle Boot Camp to receive a study gift card at the end of the training period and to avoid doing undesirable activities, like homework. This excitement waned over time.

“At first I got excited by the gift card. I got excited, and then when I had do it everyday, I got not as much excited.” [Child 1].

Parental reminders were often needed to encourage his participation in the program.

“I had to remind him everyday. I’d say like ‘today is Tuesday and Tuesday, Wednesday, Thursday is one cycle. You have to finish at least one today.’” [Parent 01].

When playing Bootle Boot Camp, the child described that the feedback version of the game made him engage with all exercises in the HEP, even those that he disliked.

“The step sideways one…I can’t skip [to the next exercise] when Coach Botley is there.” [Child 01].

#### Exercise fidelity

##### Quantitative

While four of seven prescribed exercises (i.e., hip flexion, lateral step, backwards step and single leg stance) exhibited higher exercise fidelity in the feedback condition, no significant difference was observed in overall session-level exercise fidelity between feedback (mean 0.34, SD 0.08) and no-feedback (mean 0.30, SD 0.09) conditions (*p* = 0.19) (Table 3, Online Appendix 6). Across six weeks of training, the child viewed the exercise feedback summary screen during four of his 11 feedback sessions (once during week 2, once during week 4 and twice during week 6), with a total viewing time of 3.55 min.

##### Qualitative

The child indicated that he would try to adjust his performance based on the feedback, with “all the exercises [working good] except for lateral stepping.” When the system did not count his exercise repetitions, the parent described that her child would “ask [her] for help,” prompting her to trial exercises which were inevitably tracked appropriately.

“I tried the feedback but it didn’t help…it still didn’t count it.” [Child 01].

When the child was unable to elicit different game results after adjustments were made, feedback was perceived to be incorrect and eventually ignored.

“The system feedback is not really right because it says ‘Go faster’ and then I go faster and then I get the speed warning…. I don’t listen to the feedback.” [Child 01].

In contrast, his mother felt that the feedback helped discern whether exercises were performed as prescribed, which the version without feedback could not do. While movement quality was a priority for her, she felt it was less important to him.

“I think to him, he just wants to finish the exercise. He doesn’t care whether he’s doing it right or not. He just wants to finish his work and that’s it. But for me, I want him to do it correctly. That’s the point. So I want the feedback.” [Parent 01].

This was confirmed by the child who indicated that he “didn’t really care” and “just did the exercises randomly.” He also spoke fondly about finding ways to “trick” the system into counting his movement repetitions, even when performance was not as intended.

“For the backwards stepping, I just did this [demonstrates walking backwards consecutively] instead of one and back and one and back. And it counts. It counts it so fast. It takes like 5 seconds.” [Child 01].

#### Affective engagement

##### Quantitative

Fun ratings were highly variable in both treatment conditions in the comparison phase. In the feedback condition, scores showed a steep decelerating trend towards a low level that was then maintained in the best-alone phase (Fig. 1b). The PND was 25%, reflecting only one demonstration of effect across four comparisons. Fun median scores were similar between feedback (median = 1.5, IQR = 1.8) and no-feedback conditions (median = 2.0, IQR = 1.0), with no significant difference observed (*p* = 0.53) (Table 4). Consistently low scores in the best-alone phase (i.e., median = 1.0, IQR = 0.0) were supported by the Bootle Boot Camp Acceptability Survey results where child 01 indicated a preference for the no-feedback version (Online Appendix 7). It should be noted that the full HEP was reinstated in week 4 and continued across the feedback-only best alone phase in weeks 5 and 6.

**Table 4:** Visual/statistical analysis of affective and cognitive engagement as measured through Smiley face likert rating scales

	Child 01			Child 02			Child 03		
	Comparison (4 weeks)		Best alone (2 weeks) F	Comparison (4 weeks)		Best alone (2 weeks) NF	Comparison (4 weeks)		Best alone (2 weeks) F
	F	NF		F	NF		F	NF	
*Fun ratings (affective engagement)*									
Sessions (n)	4	7	5	3	1	2	5	3	3
Level	Low	Low	Low	Low	Low	Low	High	High	High
Trend	Steep decelerating	Zero celerating	Zero celerating	Zero celerating	–	Gradual decelerating	Zero celerating	Zero celerating	Zero celerating
Variability	Highly variable	Highly variable	Stable	Stable	–	Somewhat variable	Stable	Stable	Stable
PND (%)	25.0		n/a	0.0		n/a	0.0		n/a
Median	1.5	2.0	1.0	2.0	2.0	1.5	5.0	5.0	5.0
IQR	1.0–2.8	1.5–2.5	1.0–1.0	2.0–2.0	2.0–2.0	1.2–1.8	5.0–5.0	5.0–5.0	5.0–5.0
p-value^+^	0.53		n/a	–		n/a	–		n/a
*Helpful for body ratings (cognitive engagement)*									
Sessions (n)	4	7	5	4	2	2	5	3	3
Level	Moderate	Moderate	Low	Moderate	Low	Moderate	High	High	High
Trend	Steep decelerating	Gradual decelerating	Zero celerating	Gradual accelerating	Steep accelerating	Gradual accelerating	Zero celerating	Zero celerating	Zero celerating
Variability	Highly variable	Highly variable	Stable	Stable	Moderately variable	Moderately variable	Stable	Stable	Stable
PND (%)	50.0		n/a	50.0		n/a	0.0		n/a
Median	2.5	3.1	1.0	3.0	2.0	3.5	5.0	5.0	5.0
IQR	1.0–4.2	1.5–3.0	1.0–1.0	3.0-3.2	1.5–2.5	3.2–3.8	5.0–5.0	5.0–5.0	5.0–5.0
p-value^+^	0.37		n/a	0.26		n/a	–		n/a

F = feedback, N = no feedback

^a^Fun smiley face ratings ranging from 1 = no fun at all to 5 = lots of fun

^b^Helpful for the body smiley face ratings ranging from 1 = not helpful for the body to 5 = very helpful for the body

^+^Approximate two-sample Fisher-Pitman permutation test was used to assess if the median differences in level of fun and helpful for body ratings were statistically significant in cases with unpaired data (unequal number of feedback and no feedback sessions), with a level of significance of 0.05 applied

– Indicates that the value could not be calculated based on limited data and/or a lack of variance

##### Qualitative

Failures in movement tracking on feedback days and repetitive cues related to a speed tracker safety feature led to significant feelings of anger and frustration for child 01 and his parent.

“And the speed warning. It kept coming up, even though I’m not, I’m moving so slowly…I couldn’t even do one exercise without that reminder. It made me feel kind of angry. I kind of rage quit the machine.” [Child 01].

“Sometimes when I was so busy in the kitchen, he would ask me to do the exercise to see if the computer counts my movements. Made me feel annoyed too.” [Parent 01].

When asked about this in relation to his survey results, the child indicated that he “just randomly filled [the survey] out because there were so many questions,” however confirmed that he preferred playing Bootle Boot Camp without feedback. He described game play as “just wanting to do it and get it over with.”

#### Cognitive engagement

##### Quantitative

Helpfulness for the body scores were highly variable in both conditions during the comparison phase, with decelerating trends (Fig. 1c; Table 4). Scores stabilized to a low level in the feedback condition that was maintained in the feedback-only best alone phase (Fig. 1c). The PND was 50%, reflecting two demonstrations of effect across four comparisons. Median helpfulness for the body scores were similar between feedback (median = 2.5, IQR = 3.2) and no-feedback conditions (median = 3.1, IQR = 1.5) with no significant difference observed (*p* = 0.37) (Table 4). Consistently low helpfulness scores in the best alone-phase (i.e., median = 1.0, IQR = 0.0) were supported by survey results where child 01 indicated that he did not feel the feedback helped him perform exercises better (1.0/5.0) or learn new exercises (2.0/5.0) (Online Appendix 7).

##### Qualitative

The child indicated that while he perceived exercise videos as being helpful because “they teach you how to do the exercises,” in general he did not feel either game version was helpful for his body. Coach Botley was described as “not helpful.”

His parent on the other hand, felt that both versions of the game were helpful based on the exercises being matched to her child’s needs and abilities, with feedback additionally contributing to understanding whether exercises were performed as intended.

“I think the one with the Coach is helpful, although not much difference, but I think with the Coach it will tell you right from wrong.” [Parent 01].

Feedback was particularly valued within the game because it was typically absent from HEP.

“I think the PT never gives us feedback because she just tells us what to do at home. And then she never checks how, if you’re doing it right or not…. There’s no feedback normally.” [Parent 01].

In general, the parent described that her goal was to get her son moving more by using Bootle Boot Camp, and that the game helped to achieve this. Six weeks was not perceived to be enough time to elicit any real changes to his body.

#### Overall parent and child home program experience

##### Qualitative

Overall, the parent described positive experiences with Bootle Boot Camp, recognizing its ability to support home movement practice and her child’s autonomy while minimizing the demands placed on her.

“Bootle Boot Camp seems to be more interesting because there’s music, somebody is talking in the game, so it’s not that lonely and he can do it even when I’m not there. But for the regular one [conventional HEPs], I have to look at him and ask him and push him to do it and look at him. I have to stay with him.” [Parent 01].

The child explained that he “didn’t like to do the [regular home] exercises because “it’s just boring.” Compared to his regular HEP, which the parent described as consisting of bridging and donkey kick exercises that the PT would “show us and then we would remember and do it at home,” Bootle Boot Camp was perceived to be easier to manage by both the child and parent, with the child additionally reporting that it was more enjoyable and that he felt more confident using the game when compared to his regular HEP. The parent described that her child would sometimes even “ask to do Bootle Boot Camp” of his own accord.

The child and his parent recommended several changes to optimize the gaming experience. Changes to the speed indicator were recommended to make it less repetitive and less of an interruption to exercise performance. Language used in the game was also considered to be a sensitive point when the game provided cues that the child could not respond to based on his body structure. Recommendations were made to make the language more appropriate to the movement possibilities for the child.

“It would say ‘Don’t bend your leg, don’t bend your leg.’ I can’t NOT bend my leg. It should say TRY to straighten your knee.” [Child 01].

### Phase I and II integration

Quantitative and qualitative results confirmed and expanded on each other (Table 5). Feedback helped to improve exercise adherence by encouraging the child to engage in exercise attempts. While he initially attempted to adjust his performance based on the feedback, sometimes even asking his parent for support, exercise repetitions were still not consistently counted, resulting in feedback being eventually ignored. This may explain why similarly low exercise fidelity results were observed in both treatment conditions. Repetitive feedback and technical issues led to low levels of fun, with affective engagement low for both versions of the game. Bootle Boot Camp was not a preferred activity for the child, but something he preferred doing over his homework or regular HEP. The child did not perceive feedback as being helpful, while in contrast, his parent felt that both versions were therapeutically beneficial based on the exercises prescribed by his PT, with the feedback additionally contributing to improved exercise performance.

**Table 5:** Joint display for child/parent 01 showing integrated quantitative and qualitative engagement findings with associated meta-interferences

Child parent 01				
Facet of engagement	Quantitative findings		Qualitative findings	Metainferences^a^
	Feedback	No feedback		
**Behavioural engagement**: Exercise adherence	Mean 0.98 (SD 0.24)	Mean 0.77 (SD 0.27)	*“The step sideways one…I can’t skip [to the next exercise] when Coach Botley is there.” [Child 01]* *“I tried the feedback*,* but it didn’t help…it still didn’t count it.” [Child 01]*	*Confirmed (Child)*: Feedback encouraged the child to attempt his prescribed exercises by limiting his ability to skip exercises (i.e. delaying the appearance of the ‘Next Exercise’ button), counting exercise repetitions, and by providing three extra repetition attempts beyond what was prescribed to promote optimal performance. This led to higher exercise adherence.
	*p* < 0.001^+^			
**Behavioural engagement**: Exercise fidelity	Mean 0.34 (SD 0.08)	Mean 0.30 (SD 0.09)	*“The system feedback is not really right because it says ‘Go faster’ and then I go faster and I get the [speed] warning.” [Child 01]* *“…he would ask me to do the exercise to see if the computer counts my movements.” [Parent 01]* *“I don’t listen to the feedback.” [Child 01]* *“I think to him*,* he just wants to finish the exercise. He doesn’t care whether he’s doing it right or not. He just wants to finish his work. But for me*,* I want him to do it correctly. That’s the point. So I want feedback.”* *[Parent 01]*	*Expanded (child)*,* confirmed (parent)* Quantitative findings suggest that both versions of the game resulted in similar exercise fidelity. The child attempted to adjust his performance based on the feedback and sometimes sought out the support of his parent. However, exercise repetitions were still not counted, with feedback considered wrong and eventually ignored. Tracking limitations may have contributed to this, though performance adjustments may have been insufficient to meet acceptability criteria or the child may have been unable to meet criteria based on his body structure and function.
	*p* = 0.19^++^			
**Affective engagement** Fun smiley face ratings	Median: 1.5 / 5.0 (IQR 1.8)	Median: 2.0 / 5.0 (IQR 1.0)	*“When did you feel happy?” [Interviewer] “When there was no Coach Botley…I feel that somebody was watching me on the other side of the camera.” [Child 01* *“I don’t like Coach Botley because every time when I do it 20 times*,* it doesn’t usually count it. [Child 01]* *“[Feedback cues] were all bad. For some*,* it says ‘Go wider*,*’ like for the side to side*,* it says ‘Put your feet wider’ and ‘Go faster…’* *“When it didn’t count the movement*,* that made him frustrated*,* and he had to ask me for help. And then it began to feel kind of like a burden to him.” [Parent 01]*	*Confirmed (child)*,* confirmed (parent)* Quantitative results indicated similarly low levels of fun for both the feedback and no-feedback versions of the game, with the no-feedback version slightly preferred. This was attributable to exercise repetitions not being counted during feedback sessions, feedback cues being perceived as negative/discouraging and feelings of being “watched.” While Bootle Boot Camp was perceived to be better than homework, it was not something he enjoyed doing overall.
	*p* = 0.53^++^			
**Cognitive engagement** Helpful for body smiley face ratings	Median: 2.5 / 5.0 (IQR 3.2)	Median: 3.1 / 5.0 (IQR 1.5)	*“Which version do you feel is most helpful for your body?” [Interviewer] “Nothing.” [Child 01]* *“The Coach is not helpful.” [Child 01]* *“I thought both [versions] would be the same…if the exercise is all good for his problem*,* that would be helpful.” [Parent 01]* *“I think the one with the Coach is helpful*,* although not much difference*,* but I think with the Coach it will tell you right from wrong.” [Parent 01]*	*Confirmed (child*,* parent)* Quantitative and qualitative results indicated that the child perceived feedback as not being helpful for his body, suggesting that both versions of the game were equivalent. For his parent, both versions were considered helpful based on the prescribed exercises being matched to his needs and abilities, with feedback additionally valuable for exercise performance.
	*p* = 0.37^++^			

^a^Metainferences classified as confirmed (quantitative and qualitative findings agree), discordant (findings disagree) or expanded (expands understanding of findings)

^+^Single-case randomization test (SCRT) was used to determine if the mean difference in exercise adherence was statistically significant, with a level of significance of 0.05

^++^Approximate two-sample Fisher-Pitman permutation test was used to assess if mean difference in exercise fidelity and median differences in level of fun and helpful for body ratings were statistically significant. A level of significance of 0.05 was applied

## Child-parent dyad 02

Child 02 set three lower limb goals with her PT: (1) improve ankle range of motion mobility for standing, running and stair climbing, (2) improve endurance for walking long distances, and (3) reduce falls. She was prescribed one set of 5 exercises (i.e., sit to stand, backwards stepping, forward step, supported calf stretch and seated hamstring stretch) for each session. During her initial home onboarding session, technical issues were experienced that prevented session completion. The program was stopped to enable game refinements, with the 6-week training program restarted one week later.

The child completed 6 of 16 exercise sessions and 4 smiley face ratings across these 6 sessions during the comparison period. Mean active play time was 4.31 min (SD 0.4 min) in feedback sessions and 5.08 min (SD 0.04 min) in the no-feedback sessions. Based on the mean proportion of prescribed exercise repetitions attempted, no-feedback was used during the best-alone phase. During this phase, 4 of 8 exercise sessions were completed, with a mean active play time of 4.56 min (SD 0.73 min).

### Behavioural engagement

#### Exercise adherence

##### Quantitative

**Fig. 2:**
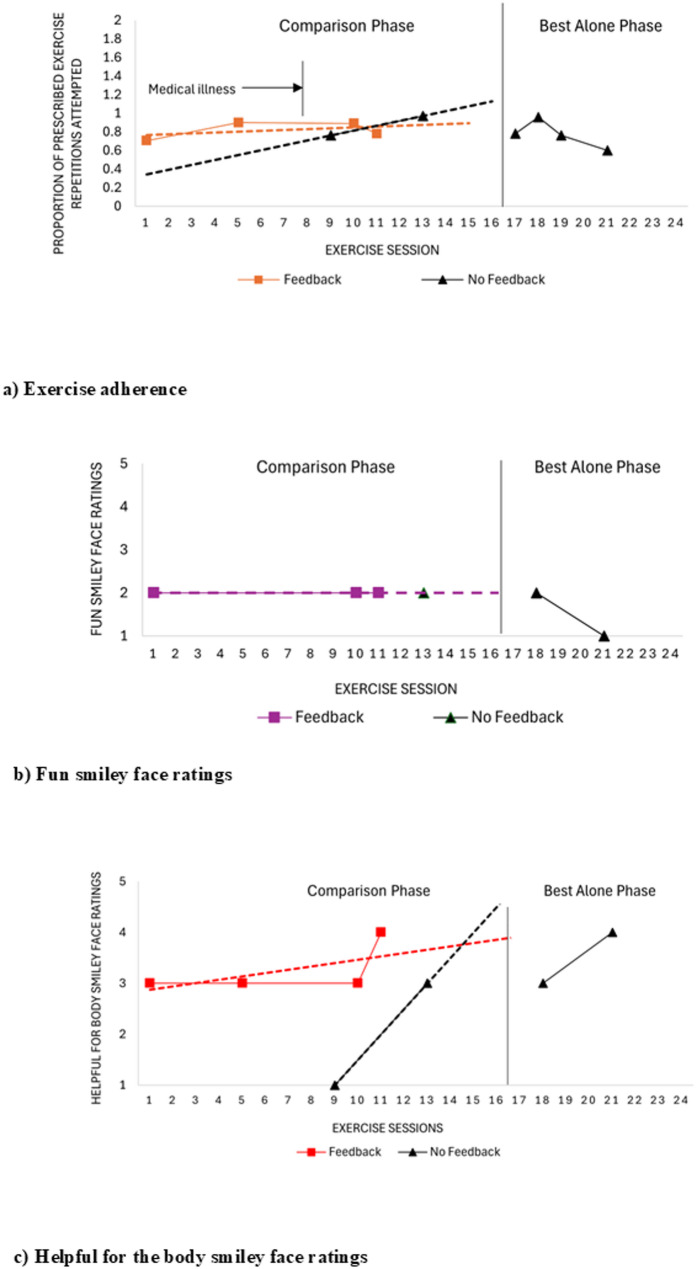
Engagement outcomes for child 02. **a** Exercise adherence (i.e. proportion of prescribed exercise repetitions attempted) as a measure of behavioural engagement, where a value of 1.0 indicates completion of all prescribed exercise repetitions and sets across exercises and a value greater than 1.0 indications completion of more exercise repetitions than what was prescribed. **b** Fun smiley face ratings (i.e. affective engagement) across exercise sessions where 1 = no fun at all and 5 = lots of fun. **c** Helpfulness for the body smiley face ratings (i.e. cognitive engagement) across exercise sessions where 1 = not helpful for the body and 5 = very helpful for the body. No technical issues were experienced for child 02 when the exercise program was reinitiated

While overall program adherence (i.e., completing scheduled exercise sessions) was low, exercise adherence within the sessions completed was consistently high across both treatment conditions, with no trend observed in the feedback condition (Fig. 2a; Table 3). For the no-feedback condition, a gradual accelerating trend during the comparison phase changed to a gradual decelerating trend in the best-alone phase, with this change in data patterns potentially indicative of multitreatment interference. The PND was 0% across two comparisons made. Adherence was similar for the feedback (mean 0.82 SD 0.09) and the no-feedback condition (mean 0.86, SD 0.15), with moderate evidence in favour of no difference between treatment groups (BF = 0.62). Adherence in the no-feedback best-alone phase was 0.78 (SD 0.15).

##### Qualitative

This child’s overall program adherence was impacted by a medical condition in the first 2 weeks of the study; however, this was not reported to the research team until the post-study interview. As her health status improved, her engagement with the program increased. The mother further described her child as experiencing anxiety related to HEPs in general, and while the family had to “push a little harder” to get her exercising with Bootle Boot Camp initially, this improved over time.

“She became more engaged as time passed, mostly because she began to really understand what the program was about and that there really wasn’t that external judgement. She just then worked through it with ease probably around the third week.” [Parent 02].

As per the parent’s comments, feedback within the game did not appear to impact the child’s adherence, with both versions described as “comparable.”

#### Exercise fidelity

##### Quantitative

Three of 5 exercises prescribed (i.e., backwards stepping, forward step and calf stretch) demonstrated higher movement quality in the feedback condition, however no significant difference was observed in overall session-level exercise fidelity between feedback (mean 0.34, SD 0.16) and no-feedback (mean 0.30, SD 0.08) conditions (*p* = 0.46) (Table 3, Online Appendix 8). The child did not use the feedback exercise summary on any occasion.

##### Qualitative

Adjustments made in response to the feedback were felt to improve exercise performance.

“She would adjust. She would try and adjust to what was being asked of her. For the better.” [Parent 02].

In some cases, the child did not know how to perform certain exercises but did not use exercise instructions within the game to support her learning. The parent went on to describe that visual and audio feedback can be difficult for her daughter to understand, with tactile feedback more beneficial for her exercise performance.

“She has a mild intellectual disability, so sometimes having her feel the movement, the correct movement is helpful where the PT would be physically correcting how she’s working through the exercise. Versus just being told or watching it, it’s a little more challenging for her.” [Parent 02].

The mother reflected that while the movement tracking appeared to be accurate, feedback did not always seem to follow suit.

“It tracked her well, like the little lines were proper where they were supposed to be and all of that. But it wouldn’t always count every single repetition as a repetition…. where it seemed to me that the form was pretty good.” [Parent 02].

#### Affective engagement

##### Quantitative

Fun was consistently rated by the child as low in both treatment conditions, with no trend observed during the comparison phase and a decelerating trend observed in the no-feedback best-alone phase (Fig. 2b; Table 4). The PND was 0% for the one comparison that could be made. No difference in perceived fun was identified (median = 2.0, IQR = 0.0) for both treatment conditions (Table 4),however a formal statistical test could not be performed due to the limited data and lack of variance. Low fun scores (i.e., median = 1.5, IQR = 0.6]) were also observed in the best-alone phase, with no survey data available to corroborate these findings.

##### Qualitative

The mother indicated that movement feedback did not have a significant impact on her daughter’s emotional involvement with the game or overall training experience.

“I don’t think there was a huge difference.” [Parent 02].

She did note though that when exercise repetitions were not counted by the system on feedback days, this often led to discouragement, with the family helping to support the management of the associated technical issues.

“It didn’t always register that she was completing what was being asked – the exercise. So that was frustrating for her. When we were here, we would just count and when she got to 10, we would just move on. That was pretty stressful for her.” [Parent 02].

The parent went on to describe that such issues were to be expected during a research trial and were not particularly problematic.

“I wasn’t overly concerned. [Technical issues] were corrected pretty quickly. And knowing that it’s a trial, we knew that there could be bumps in the road. Yeah, it wasn’t, it was fine.” [Parent 02].

Rewards feedback in the game was also not seen as motivating for the child, with the parent describing it as “prolonging the time she had to sit there and work through the program.”

#### Cognitive engagement

##### Quantitative

Helpfulness for the body scores were moderate and gradually accelerating in the feedback condition, while starting low and steeply accelerating in the no-feedback condition (Fig. 2c). The PND was 50%, reflecting one demonstration of effect across two comparisons. Median helpfulness scores were higher in the feedback condition (median = 3.0, IQR = 0.2) as compared to the no-feedback condition (median = 2.0, IQR = 1.0), but this difference was not significant (*p* = 0.26) (Table 4). Moderate scores were observed in the best-alone phase (median = 3.5, IQR = 0.6), with no supplementary survey data available.

##### Qualitative

The parent described that using the game to exercise in general was helpful, rather than a specific version of the game. She anticipated that Bootle Boot Camp would help get her child moving and stronger ahead of scheduled orthopedic surgeries, but that big functional changes were not expected because her daughter was already very active. She went on to explain that she anticipated the game would be beneficial for motor learning and strengthening through repetitive practice.

“I did anticipate that things like sit to stand would become a little easier. A lot too because of so many repetitions, it’s then you know the motor memory but also increasing her strength.” [Parent 02].

#### Overall parent and child home program experience

##### Qualitative

The parent described that her daughter has difficulties adhering to her regular HEP.

“She would do long sitting for half an hour. It was supposed to be three to four times a week. We met with the PT and she went through all the exercises with us, made sure we knew what to do, how to do it. We might have been successful once. We would attempt to do it more often but she wouldn’t…she would choose not to do it.” [Parent 02].

In contrast, playing Bootle Boot Camp was seen to be a much more positive experience for her, with the child able to play the game without the support of her family.

“She could go and do it on her own. And it was never a problem…. She definitely enjoyed Bootle Boot Camp more than the regular physio that she’d be doing.” [Parent 02].

The mother went on to explain that if given the opportunity, she would like her child to continue to use Bootle Boot Camp.

“She has performance anxiety and not having somebody there dictating what’s going on and watching her really helped. She was able to then, you know, kind of go at her own pace, and there wasn’t that external pressure.” [Parent 02].

With respect to game refinements, this parent recommended having additional time added to exercise transitions. To optimize learning, she proposed having multiple exercise views including a front and side view. She also felt that having the ability to see changes in her child’s exercise performance across several weeks would be beneficial for herself and the PT to see and control, with a phone application suggested.

“Her form, if her form is improving. Or sit to stands being timed, how that looks across the board for six weeks.” [Parent 02].

### Phase I and II integration

Qualitative data confirmed and expanded on quantitative results (Table 6). Bootle Boot Camp in general helped remove barriers to exercise for this child, enabling her to complete her prescribed exercise repetitions and resulting in similarly high levels of exercise adherence with both versions of the game. Feedback was perceived to be helpful for exercise performance by her parent; however, it was regarded as sometimes not being understood and perhaps ignored, potentially explaining why significant differences in fidelity were not observed between treatment conditions. When repetitions were not counted, this contributed to transient feelings of discouragement, but by and large, feedback did not impact the child’s affective engagement. Both versions of the game were seen as equally helpful as each promoted repetitive home movement practice.

**Table 6:** Joint display for child/parent 02 showing integrated quantitative and qualitative engagement findings with associated meta-interferences

Child parent 02				
Facets of engagement	Quantitative findings		Qualitative findings	Metainferences^a^
	Feedback	No feedback		
**Behavioural engagement**: Exercise adherence	Mean: 0.82 (SD 0.09)	Mean: 0.86 (SD 0.15)	*“Do you feel that she kept up with one version of the game more than the other or was it comparable?” [Interviewer] “No*,* it was comparable.” [Parent 02]* *“Once she got into it with each session*,* she did well.” [Parent 02]*	*Confirmed (parent)* Both versions of Bootle Boot Camp enabled the child to complete her exercise plan in alignment with what had been prescribed by the physiotherapist, leading to similarly high levels of exercise adherence.
	BF = 0.62^+^			
**Behavioural engagement**: Exercise fidelity	Mean: 0.34 (SD 0.16)	Mean: 0.30 (SD 0.08)	*“She would adjust. She would try and adjust to what was being asked of her…for the better.” [Parent 02]* *“The forward step. And then the calf stretch. They appeared very similar. And we couldn’t remember exactly the movement for each and they just looked the same and neither would register. So she just did whatever and moved on.” [Parent 02]* *“I don’t know that she even really listened to [the feedback] honestly.” [Parent 02]* *“She has a mild intellectual disability*,* so sometimes having her feel the movement*,* the correct movement is helpful…versus just being told or watching it*,* it’s a little more challenging for her.” [Parent 02]* *“It wouldn’t always count every single repetition as a repetition. Where it seemed to me that the form was pretty good.” [Parent 02]*	*Expanded (Parent)* Quantitative results suggest that movement feedback may have contributed positively to exercise performance for some exercises, with qualitative findings from the parent confirming this. Feedback may not have been helpful for all exercises because the child may not have understood the feedback or ignored it. In other cases, quality criteria may have exceeded this child’s motor capabilities, which may explain why no significant difference in exercise fidelity between treatment conditions was observed. Customizable movement acceptability criteria may be needed that are matched to each child’s needs and abilities.
	*p* = 0.46^++^			
**Affective engagement** Fun smiley face ratings	Median: 2.0 / 5.0 (IQR 0.0)	Median: 2.0 / 5.0 (IQR 0.0)	*“Well*,* it kind of*,* [the movement feedback] was slightly discouraging for her now that I’m thinking about it when it would tell her to ‘Slow down’ but she could have just been getting into position.” [Parent 02]* *“Which version do you think [child 02] preferred?” [Interviewer] “Probably the one with no feedback. But I mean*,* I don’t think there was a huge difference.” [Parent 02]*	*Confirmed (parent):* While feedback cues being provided at inappropriate times resulted in transient feelings of discouragement, feedback did not significantly impact the child’s overall affective engagement.
	p n/a			
**Cognitive engagement** Helpful for body smiley face ratings	Median: 3.0 / 5.0 (IQR 0.2)	Median: 2.0 / 5.0 (IQR 1.0)	*“Did you find one version more helpful for her body or were they comparable?” [Interviewer]* *“They were comparable.” [Parent 02]* *“I did anticipate that things like the sit to stands and whatnot would become a little bit easier…. because of so many repetitions*,* it’s then you know the motor memory*,* for her…but also increasing her strength.” [Parent 02]*	*Confirmed (Parent)*: Both versions of the game were regarded as being equally helpful for the body by promoting repetitive movement practice that was perceived to be beneficial to the child’s motor learning and overall strength.
	*p* = 0.26^++^			

BF = Bayes Factor

^a^Metainferences classified as confirmed (quantitative and qualitative findings agree), discordant (findings disagree) or expanded (expands understanding of findings)

^+^Bayesian t-test was used to determine if mean difference in exercise adherence was statistically significant

^++^Approximate two-sample Fisher-Pitman permutation test was used to assess if the mean difference in exercise fidelity and median difference in level of helpful for body ratings were statistically significant. A level of significance of 0.05 was applied

## Child-parent dyad 03

Child 03 set three lower limb goals with her PT: (1) to be able to make her bed, (2) to be able to stand in the shower, and (3) to be able to walk up/downstairs at home safely with no railings. She was prescribed 2 sets of 4 repetition-based exercises (kicking, sit to stand, seated star jump, and lateral step) and 3 sets of 2 time-based exercises (tandem stance and forward step). After the first exercise session, the family requested to have the exercise plan shortened, with the plan reduced to one set of each exercise in consultation with her PT.

This child completed 12 of 16 exercise sessions during the comparison period and 8 smiley face ratings, with a mean active play time of 4.28 min (SD 2.07) min during feedback sessions and 3.33 min (SD 1.67 min) during no feedback sessions. During week 2, child 03’s parent notified the research team of a family trip over the Thanksgiving weekend that would prevent her child from completing one of the weekly scheduled sessions. Upon their return, exercise repetitions were limited by technical issues that were caused by the device being unplugged. These were addressed by the research team by the start of week 3. During the first three sessions in week 4, the child used the multiplayer function of the game, with a family member as her play partner. Based on the mean proportion of prescribed exercise repetitions attempted, the feedback version was used in the best-alone phase. In this phase, the child completed 4 of 8 exercise sessions, however a system error resulted in a no-feedback session being mistakenly provided. A mean active play time of 3.37 min (SD 0.51) was observed across the 3 feedback-based exercise sessions. Technical issues experienced during week 6 prevented completion of the last two sessions.

### Behavioural engagement

#### Exercise adherence

##### Quantitative

**Fig. 3:**
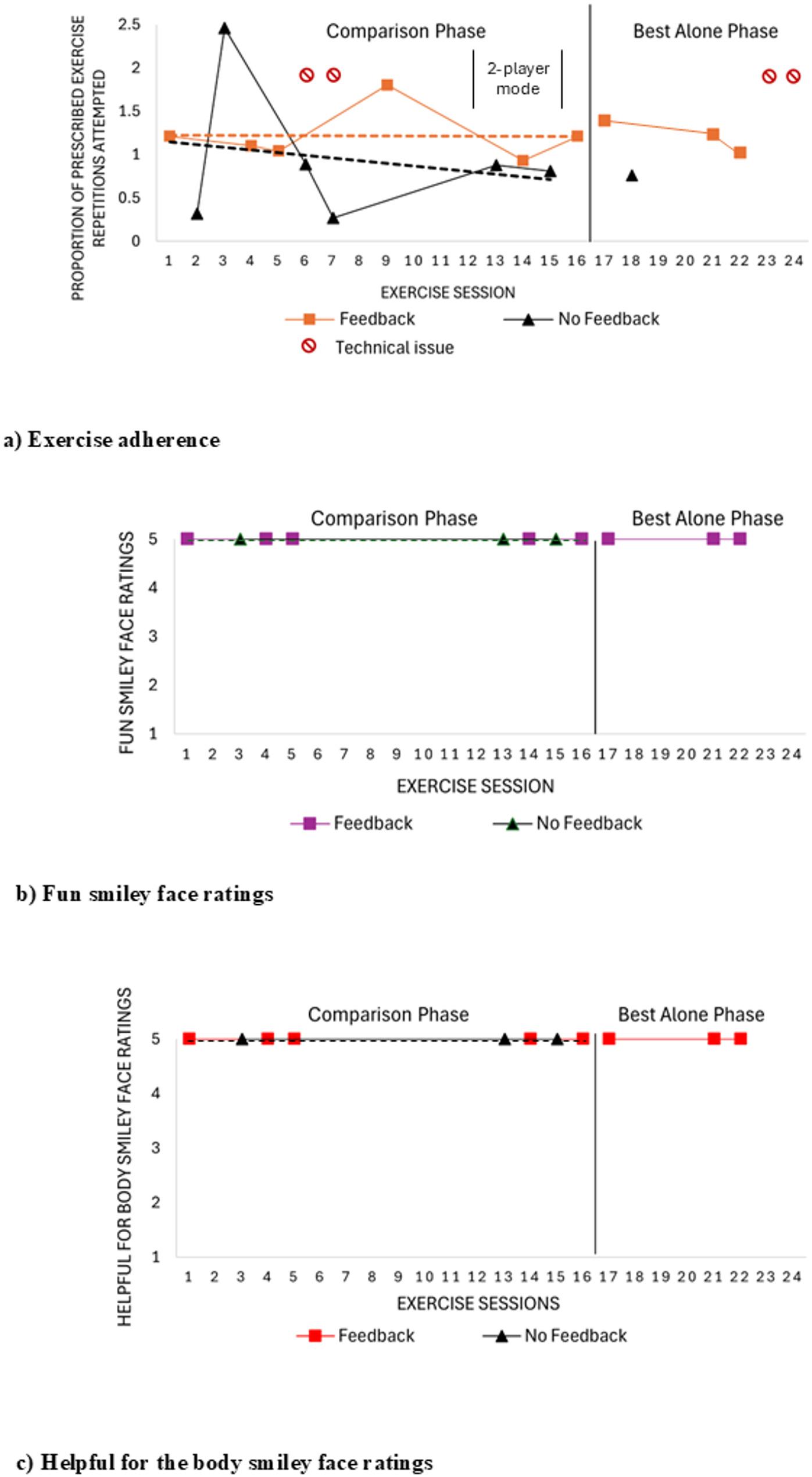
Engagement outcomes for child 03. **a** Exercise adherence (i.e. proportion of prescribed exercise repetitions attempted) as a measure of behavioural engagement, where a value of 1.0 indicates completion of all prescribed exercise repetitions and sets across exercises and a value greater than 1.0 indications completion of more exercise repetitions than what was prescribed. **b** Fun smiley face ratings (i.e. affective engagement) across exercise sessions where 1 = no fun at all and 5 = lots of fun. **c** Helpfulness for the body smiley face ratings (i.e. cognitive engagement) across exercise sessions where 1 = not helpful for the body and 5 = very helpful for the body

Exercise adherence was consistently higher in the feedback condition, except for one no-feedback session in which this child completed more than double the prescribed number of exercise repetitions (session #3) (Fig. 3a). This was maintained in the feedback-only best alone phase. The PND was 83.3%, reflecting five demonstrations of effect across six comparisons. The child achieved a mean exercise adherence of 1.21 (SD 0.30) in the feedback condition compared to 0.94 (SD 0.80) in the no-feedback condition, but this was not statistically significant (*p* = 0.57) (Table 3). In the single no-feedback session that was mistakenly delivered in the best-alone phase, exercise adherence was lower than all feedback sessions in this phase.

##### Qualitative

This parent described that while her child needed reminders to complete her exercise sessions, her motivation to play the game was high. The child reinforced these sentiments, describing how feedback helped encourage her participation.

“I like Coach Botley so much. Coach Botley gives me instructions and stuff like ‘Let’s get started’ and stuff like that…. Sometimes I see the Coach and I just watch the Coach. But I also watched the exercises and I do it. I like the exercises and the most thing I like is the stars and awards and stuff. If they don’t give me stars, I’m totally not going to do it if they don’t count it.” [Child 03].

She went on to describe that feedback from Coach Botley helped improve her self-confidence.

“The Coach normally makes me feel like I can do anything.” [Child 03].

Despite this preference for feedback, the child was observed to complete more than double the prescribed exercise repetitions during a no-feedback session. When asked about this, she demonstrated a lack of awareness and familiarity with the system.

“I was counting before I do the next exercise. I only did 10 and then I moved on…I was not really used to the game.” [Child 03].

This child’s motivation was perceived by her parent to slightly decline when she experienced a fall in week 5 after setting up the game and mistaking the position of a chair when sitting down. This was not reported to the research team; however, the parent described the event in the interview, explaining that the child “did not have any injuries from Bootle Boot Camp.” The child suggested that it “didn’t change anything” and “was kind of funny.”

#### Exercise fidelity

##### Quantitative

While exercise fidelity was higher for all six exercises when feedback was enabled, the overall difference between session-level fidelity between the feedback (mean 0.71, SD 0.14) and no-feedback condition (mean 0.35, 0.08) was not statistically significant (*p* = 1.0) (Table 3, Online Appendix 9). The child reviewed her exercise feedback summary on 8 of 9 possible feedback sessions during her 6-weeks of training (total viewing time of 3.41 min).

##### Qualitative

The parent regarded feedback as helping improve her child’s movement quality but recognized the limitations of technology providing this feedback over a clinician.

“When on those feedback days, I think Botley was really good at making sure she’s standing tall and aligned and kind of in the frame, and that was helpful. But obviously it’s different because while he’s recommending stuff in 2D, her physiotherapist can see everything.” [Parent 03].

Review of exercise videos during feedback days in which multiplayer mode was used (week 4) revealed that while the child did not always respond to feedback cues on screen (e.g., continued to perform lateral step exercise incorrectly), a family member playing with her would respond, helping to optimize her performance by providing additional demonstrations and cues which led to better performance that persisted in subsequent sessions. When asked about this however, the child indicated that she did not feel that playing with a family member changed her exercise performance.

#### Affective engagement

##### Quantitative

Fun scores were high in both treatment conditions with no trends observed (Fig. 3b). The PND was 0% for the three comparisons made. Median fun scores were 5.0 (IQR = 0.0) for both treatment conditions (Table 4). A formal statistical test could not be performed due to the lack of variance in the data (i.e., all sessions rated 5.0). In the feedback-only best alone phase, a median fun score of 5.0 (IQR = 0.0) was observed. Survey results revealed that child 03 preferred Bootle Boot Camp with feedback (Online Appendix 7).

##### Qualitative

The mother explained that Bootle Boot Camp helped improve her child’s enjoyment of exercise.

“I think it was the initial surveys that we did for this where she indicated over and over again in the test that she did not like physical activity. So that was kind of a change in [child’s name] perception of exercising. But it was also nice to see it in action and how she enjoyed Bootle Boot Camp.” [Parent 03].

The opportunity for social play helped to maintain the child’s positive emotional involvement with the game. The child described that playing with a family member was “more fun than playing alone.” Her parent reflected that multiplayer mode enabled the child to interact with more game features and helped sustain the child’s excitement.

“They were buying stuff from the little store. So that was really exciting for them I think. [Child’s name] hadn’t discovered that before. Or she wasn’t using the feature, maybe she forgot about it. But with [her family member’s] presence, they were using the money and it was really motivating for her to discover this other feature that she didn’t know about. And making money, the robot bucks, she was super excited to get that at the end and knew what she could do with it.” [Parent 03].

This child and her parent described a strong preference for the feedback game version. However technical issues, including game freezes and movement tracking issues caused “a lot of frustration” as reported by her parent. These feelings were mitigated by timely technical support, which was highly valued.

“I thought that was like a lot of involvement that you’re picking up almost live, so that was really comforting too that you’re reviewing as we go rather than just at the very end.” [Parent 03].

#### Cognitive engagement

##### Quantitative

Helpfulness for the body scores were high in both treatment conditions with no trends observed (Fig. 3c). The PND was 0% for the three comparisons made. Median helpfulness scores were 5.0 (IQR = 0.0) for both conditions (Table 4). A formal statistical test could not be performed due to the lack of variance in the data (i.e. all sessions rated 5.0). A median fun score of 5.0 (IQR = 0.0) was observed during the feedback-only best alone phase and was supported by survey results that showed that child 03 perceived feedback as being helpful for exercise performance (5.0/5.0) and learning new exercises (5.0/5.0) (Online Appendix 7).

##### Qualitative

The child indicated that feedback was helpful for understanding how to perform exercises, making training easier overall.

“[Exercising] was easier if it gives me that instruction. It was easy to do, from the instruction.” [Child 03].

The parent described that her child was able to achieve her therapy goals, which she credited directly to the game.

“When she set the goal of ‘Okay I want to do my bed, I want to walk up stairs, I’m going to stand in the shower,’ all of them in my head unfortunately, I was like ‘Yeah it’s not going to happen.’ But two of those three goals, she’s been doing them very consistently. She makes her own bed now. She walks up and down the stairs. She exceeded my expectations…I think it’s directly related to the game.” [Parent 03].

### Overall parent and child home program experience

**Quantitative** The child described that as part of her regular HEP, which included “keeping a ball between the knees” and “moving in a box from the left and high fiving,” her PT showed her and her mother the exercises and asked them to “remember them.” While the child was asked to do these exercises “as much as [she] can,” the child suggested she was not doing them because she “was being too lazy.” Her parent reflected on her daughter’s difficulties with adhering to regular HEPs, with Bootle Boot Camp helping to improve her child’s motivation to exercise.

“I thought at first, I thought, no way we’re going to do four days a week. Like we’re going from zero to four. That’s mission impossible…. And I know you have the trackers to tell us if we actually accomplished four times a week, but it felt like we were getting close or even exceeding it sometimes because Botley would tell us, ‘Well, you’re done for this week.” [Parent 03].

This was attributed to the gamified HEP format which was a welcome change for child 03.

“It’s like a game. It’s not exercise. It’s Bootle Boot Camp. We don’t say, ‘Let’s do physio now.’ ‘Let’s do Bootle Boot Camp.’ Differences in the name too, right? Because it makes a difference when these kids have been in and out of physio for so long.” [Parent 03].

The parent further explained that the game helped to reduce parental burden, as it was easy for the family to set up and for her child to use independently at times.

“Bootle Boot Camp was much easier for us because we can turn it on and she knows what to do and could do it. With the other ones, we need to set up everything physically and be there for her to switch out of. I think it was more engaging to have the application.” [Parent 03].

The parent further reflected that Bootle Boot Camp was a novel and innovative way to get her child exercising at home, helping to fill a gap in the current therapy landscape for digital therapeutic technologies for children with neurodiverse needs.

“I just think the market is lacking in terms of these type of interactive games where kids can do their exercises. And these kids are growing in a digital age where we need more of this, yet it’s not as readily available or it’s very expensive and not accessible. The fact that there is, you know innovation happening around how physio can be done. And it’s something that I’m all for.” [Parent 03].

This child and parent made recommendations for game refinements, suggesting that the no-feedback version of the game be removed. The parent also indicated that having the program progress in difficulty, based on the child’s performance and in consultation with the child’s PT, would be beneficial.

### Phase I and II integration

While quantitative and qualitative results confirmed one another with respect to behavioural engagement, discordance was noted for affective and cognitive engagement (Table 7). This child was strongly motivated by feedback related to knowledge of results (e.g., counting of exercise repetitions), knowledge of performance (e.g., star ratings) and rewards (e.g., Bootle Bucks), leading to higher adherence and exercise fidelity in the feedback condition compared to the no-feedback condition. However, while reporting that the feedback version led to higher levels of fun and perceived helpfulness for the body, this was not captured in survey ratings that were similarly high in both versions. A possible reason for this is that the child reported a response bias (i.e., selecting the most positive smiley face regardless of the treatment condition). In this case, qualitative interview data helped understand her experiences more robustly.

**Table 7:** Joint display for child/parent 03 showing integrated quantitative and qualitative engagement findings with associated meta-interferences

Child parent 03				
Facet of engagement	Quantitative findings		Qualitative findings	Metainferences^a^
	Feedback	No feedback		
Behavioural engagement: Exercise adherence	Mean: 1.21 (SD 0.30)	Mean: 0.94 (SD 0.80)	*“I like it because they sometimes count like one*,* two*,* ten. But when they don’t do that*,* I’m not going to do it unless I count to 10. And I do the next one because they’re not going to skip to the next one.” [Child 03]* *“If they don’t give me stars and stuff*,* I’m not totally going to do it if they don’t count it.” [Child 03]* *“The Coach normally makes me feel like I can do anything.” [Child 03]*	*Confirmed (child):* The feedback version promoted higher exercise adherence as the child was highly motivated by knowledge of results (e.g., repetition counter) and knowledge of performance/rewards feedback (e.g., star ratings based on proportion of exercise repetitions completed with exercise fidelity) that were not present in the no-feedback game version.
	*p* = 0.57^+^			
Behavioural engagement: Exercise fidelity	Mean: 0.71 (SD 0.14)	Mean: 0.35 (SD 0.08)	*“Did you feel like you were doing your exercises better with Coach Botley?” [Interviewer] “Yes.” [Child 03]* *“What about when the Coach was telling you to maybe take a bigger step? Or to stand tall? Did you find that helpful?” [Interviewer] “It was like easy to do from the instruction.” [Child 03]* *“The feedback about standing tall and making sure she’s aligned… all of that was beneficial. [Parent 03]*	*Confirmed (child, parent):* Movement feedback was valuable to achieving higher exercise fidelity, as confirmed by qualitative findings from both the child and parent. Feedback cues and instructions helped improve the child’s understanding of appropriate movement performance and competence to move and exercise.
	*p* = 1.0^++^			
Affective engagement Fun smiley face ratings	Median: 5.0 / 5.0 (IQR 0.0)	Median: 5.0 / 5.0 (IQR 0.0)	*“With Coach Botley is more fun.” [Child 03]* *“I liked Coach Botley so much. Because Coach Botley gives me instructions and stuff like*,* ‘Let’s get started.’” [Child 03]* *“I see the Coach and I just watch the Coach. But I also watched the exercise*,* and I do it. But I like the exercises and the most thing I like is the rewards and awards and stuff.” [Child 03]* *“Yeah*,* I heard her say ‘I don’t know what to pick.’ Like just didn’t know how she felt about it to pick. She’d go for the furthest one*,* the happiest one.” [Parent 03]*	*Discordant (child, parent):* Despite the child describing a strong preference for the feedback game version, fun ratings were the same for both versions of the game, suggestive of no difference. This may have been because the child was selecting the most positive smiley face ratings rather than ratings which accurately reflected her feelings. Incorporating multiple data sources and using observational and interview data may be important to better understand child participants’ exercise experiences.
	p n/a			
Cognitive engagement Helpful for body smiley face ratings	Median: 5.0 / 5.0 (IQR 0.0)	Median: 5.0 / 5.0 (IQR 0.0)	*“The one without [the feedback] is a little bit helpful*,* but with the robot is more helpful.” [Child 03]* *“So when you were answering those smiley face ratings*,* were you thinking about which face you were picking or just picking one at random?” [Interviewer]* *“I’m just picking one at random. I don’t know which one*,* and then I just picked one.” [Child 03]* *“The counting [in the feedback version] was definitely very helpful for her. I think for her*,* number one was the counting.” [Parent 03]*	*Discordant (child, parent):* While both the child and parent felt that the feedback version of the game was more helpful for the child’s body, ratings did not reflect this and were suggestive of no difference between game versions. This may be because the child indicated that she was selecting smiley face ratings “at random” rather than making selections based on her true beliefs. Incorporating multiple data sources and using observational and interview data may be important to better understand child participants’ exercise experiences.
	p n/a			

^a^Metainferences classified as confirmed (quantitative and qualitative findings agree), discordant (findings disagree) or expanded (expands understanding of findings)

^+^Single-case randomization test (SCRT) was used to determine if mean difference in exercise adherence was statistically significant, with a level of significance of 0.05

^++^ Approximate Wilcoxon-Pratt Signed-rank test was used to determine if median difference in exercise fidelity was statistically significant. A level of significance of 0.05 was applied

## Discussion

This study aimed to understand the impact of movement-tracking feedback in an ICP-HEP on multiple facets of children’s engagement and children’s and parents’ experiences using a multi-case mixed methods study design. We had initially intended to explore this relationship with 3–4 children per age and gender stratum (16 children total). However, our identification of movement-tracking limitations in some children, exercises, and play environments, as well as recommended game refinements by study participants prompted us to stop further enrollment to investigate tracking issues, explore different types of movement-tracking technologies, update movement metrics (e.g., potential for customizable metrics for each exercise), and home onboarding and training processes to optimize play experiences. The plan will then be to move back to a larger-scale trial with the revised product to allow us to resume the study with the original objectives. Although this is not what we had anticipated, it exemplifies our iterative development, testing and prototype refinement processes, as outlined in the design thinking framework [34, 35]. We hope that sharing such information will help other clinical research teams avoid similar pitfalls and bring awareness to the developmental importance of collaborative and iterative design processes in real world settings. Our reliable approach of manually reviewing exercise repetition attempts and quality repetitions enabled us to still interpret movement data and engagement outcomes with these first three children, with the integration of quantitative and qualitative data providing a rich understanding of these children’s and parents’ experiences with feedback and Bootle Boot Camp at large. Given that movement-tracking and resultant feedback were not always appropriate, we present the following key findings related to each study objective for these first three child-parent dyads as early-stage evidence and subject to further validation.

## Behavioural engagement (Objective 1)

*Key finding #1: Feedback may have supported exercise adherence for two children*,* without negatively impacting adherence for the other child.*

The impact of feedback on exercise adherence was somewhat positive for child 01 and 03, with the extent of this improvement reaching statistical significance for child 01. This adherence impact was in part due to feedback mechanisms that encouraged all children to trial rather than skip exercises within the program (e.g., repetition counter), with child 03 additionally motivated by performance and rewards feedback (e.g., star ratings). For child 02, exercise adherence was comparable in both versions and remained high when feedback was used, with the game in general enabling completion of the prescribed exercises within her HEP. Overall program adherence was mediated by extrinsic factors for all three children, including parental reminders, the presence of family members, and the promise of real-world rewards (e.g., gift cards). Parental reminders have been identified as a key extrinsic motivator of children’s home-based practice with therapeutic technologies, [88] with strong family support helping to facilitate participation [12, 89, 90]. 

*Key finding #2: Parents and one child perceived feedback as generally being useful for learning about movement quality. When movement feedback was perceived to be inaccurate*,* it may have been ignored.*

All children reportedly adjusted their performance based on feedback provided, which was perceived by parents to contribute to improved exercise fidelity when delivered at appropriate times. This is in line with previous studies examining the use of home-based ICP technologies with children with CP where real-time feedback helped support children’s task performance [91–93]. Children sought support from their family members when exercise repetitions were not counted. While in some cases, this was because of system tracking issues, in other instances, this was because of the child’s movement performance errors. Family members helped children to correct their exercise performance by demonstrating appropriate performance, reiterating feedback cues, and providing additional instructions. Though having another family member in view of the camera during single-player mode sometimes inadvertently resulted in the system counting and logging an incorrect number of movement repetitions (i.e., since the camera tracks the movements of the person closest to it), children and their family members worked together to try and perform exercises as prescribed. If a child remained unable to achieve high-quality repetitions after these performance adjustment efforts were made however, feedback was sometimes perceived to be wrong and thus disregarded. These factors may explain the lack of significant differences in exercise fidelity between feedback and non-feedback game versions as they resulted in four possibilities for why proper performance of an exercise did not occur in the feedback state: (1) if children were attempting to respond to feedback but could not achieve acceptability criteria because of their functional limitations, (2) their performance adjustments were insufficient to meet the movement criteria, (3) the system had technical limitations pertaining to tracking accuracy and feedback delivery, and (4) the feedback that was provided was not understood or ignored altogether. While the first two factors may signal to us that the movement criteria were too stringent for some exercises/movements, the latter two factors are indicative of system tracking/feedback issues that need to be resolved.

Clinicians have previously expressed concerns that ICP systems may be unable to correctly track performance and provide accurate and individualized feedback, with incorrect or generic feedback perceived to potentially reinforce maladaptive movement patterns [16]. Such compensatory movements during game play are perceived to have potentially negative impacts to a client’s recovery [15]. They have additionally identified that a system’s capacity to monitor performance and track compensatory movements during home-based rehabilitation is important to enable appropriate movement guidance, ensure that targeted and therapeutically beneficial movements are practiced, and provide relevant and useful feedback on movement performance, with clients perceived to care more about the movement outcome rather than movement quality when using technology [13, 14]. Our data suggest that in instances when inappropriate feedback is delivered, it may not be detrimental to performance as children may not attend to feedback they perceive to be incorrect or wrong. However, the goal will be to optimize tracking to provide the most appropriate feedback during exercise performance.

## Affective and cognitive engagement (Objective 2)

*Key finding #3: Feedback preferences varied among child*,* with technical issues leading to disengagement for all participants.*

The range of views on feedback from highly positive to indifferent or negative aligns with in-lab testing of Bootle Boot Camp where children (both neurotypical and children with CP) had varied perspectives on in-game feedback [18]. There was consensus among all children and parents in this home-based study that technical issues were particularly frustrating. While some issues were only evident to families in the feedback version of the game (e.g., movement-tracking issues that prevented quality exercise repetitions from being tracked), others were experienced in both the feedback and non-feedback version (e.g., sudden loss of sound) based on technical glitches. Some issues were easily resolved by the families (e.g., by turning the system off and back on), whereas other issues required virtual technical support from the research team via Zoom (e.g., game freezes caused by large video file sizes that resulted from the system not being turned off at the end of game play). The research team support in navigating these issues was highly valued by parents. These findings align with previous studies where technical challenges with home-based ICP systems have led to disengagement, with families benefiting from timely external technical support [12, 88, 94]. Notably, in all cases, the immediate technical support in our study helped families re-engage with the HEP, with game play resuming shortly after implementation of these technical solutions. Study recruitment was discontinued to explore how to minimize movement tracking issues at the system level (e.g., changes to movement metrics) and at the environmental level (e.g., changes to home onboarding procedures) to reduce disengagement due to technical issues.

*Key finding #4: While children had mixed perceptions on the value of the movement-tracking feedback*,* all parents reported that it was still beneficial despite its occasional informational challenges.*

Children had diverse perspectives on the value of the feedback, with one child indicating that it was not helpful as it was repetitive and frustrating, and another reporting that feedback cues helped them learn how to perform exercises. All parents perceived feedback as being beneficial for children’s learning and understanding of movement performance, particularly because this was not usually available at home. When performing home exercises without clinician supervision or feedback, children with neuromotor disorders have been found to perform exercises incorrectly and at times unsafely [3]. This reinforces previous sentiments shared by caregivers of children with CP using home-based rehabilitation technologies, in which desires for more real-time performance and progress feedback have been expressed, [12] with clinicians also highlighting the need for therapy technologies to provide positive and practical feedback to users on their movement performance [14, 15]. Future work may consider the inclusion of interview questions to probe parents on their willingness and perceived ability to take on the role of a virtual therapist (i.e., in addition to Coach Botley), as well as their preference for this to be technology-based versus parent-led. In general, Bootle Boot Camp was perceived to be valuable in terms of the exercises that were selected by the child’s PT, highlighting the important role that clinicians play in creating HEPs that are matched to the therapeutic needs, abilities and goals of each child.

## Overall child and parent home program experience (Objective 3)

*Key finding #5: Bootle Boot Camp may have enhanced the home therapy experiences for all children and parents and was reportedly preferred over conventional HEPs.*

Children and parents had a positive outlook on the use of Bootle Boot Camp to support HEPs, finding it to be a preferred and innovative way to encourage repetitive movement practice compared to their traditional HEPs. The use of gamification elements, including performance graphs, avatars and virtual rewards (e.g., badges, Bootle Bucks, stars), helped to motivate one of the three children to exercise in the absence of real-world rewards. Children were able to play Bootle Boot Camp relatively independently with no more than minimal (if any) set-up and technical support from their parent, with promotion of autonomy, competence and self-efficacy previously identified as means to support children’s intrinsic motivation with interactive technologies [95]. This further helped to reduce the onus on parents who were typically tasked with having to remember traditional home program exercise components, provide feedback on exercise performance, ensure weekly session adherence (i.e., completion of the prescribed number of home exercise sessions) and exercise adherence (i.e., completion of the prescribed number of repetitions and sets for each exercise), aligning with previous studies in which caregivers have valued interactive technologies for their contributions to reducing caregiver burden and stress [12, 94, 96]. Refinements were recommended to optimize play experiences. These included refinements to language, movement tracking, speed indicators, exercise videos, and alternative access to system-tracked performance data via a phone application.

Lessons learned: broad implications for development, evaluation and translation of interactive home therapy technologies:

1. *ICP technologies can support children’s adherence to structured HEPs*,* independent of users’ perceptions of the value of in-game feedback.* Limited adherence to HEPs is a significant concern for therapists working with children with neuromotor disorders, with adherence ranging from 34–79.2% [9, 11, 97]. This challenge was mirrored in our study results in which all families reported difficulties with or a lack of adherence to traditional HEPs. Independent of the presence or absence of feedback within the game, all children continued to use Bootle Boot Camp throughout the six-week study intervention, suggesting that interactivity and overall game features, which were designed to promote children’s intrinsic motivation through autonomy, competence and relatedness, were sufficient to maintain children’s interest with the HEP and contribute to improved program adherence. This sustained and repetitive practice of targeted movements using the ICP-HEP may help contribute to children’s learning [37]. All study participants reported a preference for using the system over their conventional HEPs, with all parents and one child finding added value in Coach Botley’s feedback, coaching cues, and rewards in relation to the quality of their exercise performance. While continued research is warranted to more fully understand the impact of movement-focused feedback on children’s exercise experiences, findings suggest that implementation of ICP technologies may support children’s participation with structured HEPs.
2. *Home-based usability testing is needed as a critical extension to lab-based studies*,* particularly in the context of movement-tracking technologies where variable environmental conditions may limit tracking accuracy. The ability to track exercise data in real-time is fundamental to successful implementation.* Most usability testing of pediatric rehabilitation technologies is conducted through single-testing sessions in the lab or hospital [98]; however favourable outcomes in the lab are not guaranteed to carry over into the home setting where families are responsible for managing systems independently [13] and environmental factors (e.g. ambient light) are more variable. In-lab testing of Bootle Boot Camp showed few technical issues (e.g., glitches, freezes) and accurate tracking of quality exercise repetitions overall when compared to the gold standard motion analysis system, with reduced (but still acceptable) accuracy noted in tracking static versus dynamic exercises, movements in the vertical dimension (compared to the medio-lateral and anterior-posterior dimensions), and tracking certain joints (i.e., ankle joint) [18, 19]. Thus, prior to home-based testing, refinements were made to the movement quality metrics to reduce reliance on joints that were identified as tracking less reliably where possible (e.g., the more easily tracked knee joint was used as proxy for the ankle joint in the tandem stance exercise). Despite these modifications, our in-home experiences revealed that exercises that tracked with high accuracy in lab (e.g., sit to stand) were also reliable in the home, while those with reduced, but still acceptable accuracy in the lab (e.g., lateral step) were below the acceptability threshold in the home. For home play overall, despite extensive parent and child orientation, diverse home environment conditions (e.g., lighting, space) as compared to the more controlled lab environment, as well as variable use conditions) reduced movement tracking reliability. While families were provided with recommendations to optimize tracking (e.g., tight-fitting clothing, playing in a clear, open and well-lit space and while standing no more than two meters from the camera), these instructions were sometimes not followed as observed through the video-recorded data. In the home environment, children were seen playing in loose pajamas, in the dark, in the presence of toys and clutter, and at variable distances from the camera. While in lab-based testing, the height of the camera and the child’s position relative to it was fixed, in home use, children were observed to move towards and away from the camera because of pets and younger siblings occasionally coming into the field of view of the sensor during exercise sessions. In some cases, the height and positioning of the camera were then adjusted by families in response. This reflects the importance of home-based testing such that system performance can be fully characterized in real-world operating conditions and measures taken to ensure adequate performance (e.g., system warnings if the child moves out of the optimal tracking zone or if playing with insufficient lighting). Rigorous usability and validation testing in the home environment should therefore be integrated into early-stage ICP-HEP system testing prior to full-scale pilot studies and clinical evaluation. We found that having real-time exercise tracking and review of exercise data to enable identification and problem solving of family-reported technical issues was valued by families. When considering future implementation of these technologies into clinical practice, the child’s therapist or a monitoring/virtual therapist may be able to fulfil this role, with clinicians indicating the need for hands-on training and experience with a technology themselves before being able to support clients’ use of these technologies [14, 16]. While for our study, the research team was able to provide this support, in future, having clinicians trial and play Bootle Boot Camp to better understand its game features, technical aspects and feedback mechanisms may be beneficial so that they can support families’ game use. Even after this training is provided, having a core technical support team may be valued as clinicians have previously expressed desires for such support to resolve challenges efficiently [14]. Reporting these technical challenges and related solutions as part of the iterative design process enhances transparency and informs future testing processes for other researchers and ICP developers [99].
3. *Mixed methods single-case research provides a methodologically robust approach for early-phase evaluation of home-based interventions*,* supporting iterative technology development.* The use of mixed methods usability testing can help give context and understanding to user challenges to guide technology refinement and future implementation efforts. A recent systematic review exploring the use of augmented reality in motor rehabilitation interventions found that qualitative methods (e.g., observation, interviews) remain largely under-applied within usability studies in this field [100], with mixed methods usability testing previously suggested as a means to holistically evaluate novel interactive technologies [101]. Combining single-subject research, that preserves and describes children’s individual outcomes and which is well suited for children with CP who have variable motor performance [48, 102, 103], with qualitative methods that describe sample and contextual characteristics [104], offers a rigorous way to conduct this stage of technology testing and iterative design [103]. This was particularly valuable for conducting research with child participants in our study, where rating responses were observed to be less reliable due to response bias and where system measured data were unable to capture medical illnesses and family trips. Integration of quantitative and qualitative data was crucial to give context to engagement outcomes and to help understand discordant quantitative rating and qualitative interview findings. Our multi-case mixed methods study design, involving a quantitative SCED phase followed by a qualitative descriptive phase, enabled us to gain valuable insights on children and parent’s home exercise experiences to continue to advance the design and implementation of our ICP-HEP.

## Limitations and future work

This study presents in-depth analyses of three child-parent dyads. Due to the limited sample size, definitive conclusions with respect to the impact and generalizability of feedback on children’s engagement outcomes are premature. However, the mixed methods nature of our study enabled us to gain rich insights and detailed narratives that enhanced the depth and relevance of our findings, while contributing strongly to our iterative development and refinement of Bootle Boot Camp. The diverse experiences of three child-parent dyads provided us with substantial information power owing to the specific, highly focused study aims, specificity of participant knowledge (i.e., extensive experience during their participation with Bootle Boot Camp), strong and focused interview dialogue with study participants, and use of established theory to guide study planning and analysis [105]. When technical refinements have been made and continued evaluation of Bootle Boot Camp in the home setting is possible, the in-depth descriptions of each child, their goals, and response to treatment interventions presented in this study may promote future transferability of findings [104]. Clinicians may then be able to use this information to determine if Bootle Boot Camp may be a reasonable fit for their clients with similar characteristics [102], based on their clients’ needs and abilities. It should also be noted that two of three child participants identified as girls and that all caregivers identified as women, with this gender representation potentially impacting study findings. Recognizing that caregivers who identify as men are often underrepresented in child studies [106], future work will aim to purposefully recruit a larger sample with greater gender representation.

While many threats to internal validity (e.g., history, maturation, instrumentation, attrition) are less likely with an alternating treatment design due to its relatively short duration, multitreatment interference is possible [46], and may have occurred in this study as indicated by changing exercise adherence trends between the comparison and best alone phases (e.g., accelerating trend changing to decelerating trend) for child 02. It is also possible that the novelty of the game may have declined over time, as has been shown in previous studies involving home-based interactive technologies [12, 107]. 

Given that serial dependency (i.e., an observed response is related to a previously observed response) is often present in single subject data [54], we used nonparametric randomization tests, which are not affected by serial dependency, and which do not rely on assumptions of normality, variance homogeneity, or specific distribution shapes [63]. The application of these tests was limited in cases with few data points however, requiring alternative approaches to be used (e.g., Bayesian t-test). Some participants also received an unequal amount of feedback to no-feedback sessions due to limited adherence and technical issues, resulting in the need to use different statistical tests to compare exercise fidelity with unpaired (Fisher-Pitman test) versus paired data (Wilcoxon-Pratt test) to prevent data loss. It should be noted that using the Fisher-Pitman test for single participant data may have violated the assumption of independence, potentially leading to biased results. Moreover, a minimum of five repetitions of the alternating sequence and five data points per condition have been recommended to meet single case design standards [108, 109], yet limited program adherence resulted in this not being met for child 02.

While the post-study survey and rating scales were developed based on established tools for measuring product acceptance and preference (e.g., Smileyometer [60, 110], This or That [60]), missing data resulting from rating scales or surveys being skipped limits our understanding of affective and cognitive engagement outcomes. Smiley face ratings further reflected scoring extremes (e.g., scores of 5 for level of fun and helpfulness for all exercise sessions for child 03), as is often the case with rating scores in younger children [60]. While the personalization of smiley face scales was done to try and improve responsiveness, reported use and related results of two of the children indicated potential biased selection of both in-session ratings and post-study survey ratings (same response option chosen through survey), with one parent additionally expressing sentiments that ratings were a “waste of time.” Our observational (e.g., review of exercise videos) and qualitative interview data enabled us to understand participant experiences more deeply. In future, the study protocol may be amended to reflect greater reliance on observational and interview data to understand children’s experiences. Ongoing partnering with knowledge holders may be warranted to identify the appropriate contexts and timing for such ratings.

Finally, movement-tracking issues variously impacted the feedback that was delivered to children, which could have negatively influenced engagement outcomes if provided inappropriately. Movement-tracking issues were multifaceted and may have stemmed from both software and hardware issues, as well as operating conditions. Research is ongoing to validate different hardware and software combinations with respect to body tracking performance. In parallel, improvements are underway for the algorithmic approaches used in Bootle Boot Camp to count exercise repetitions and deliver feedback. This will help to address children’s reports of being unable to achieve high-quality repetitions even when they adjusted their performance (e.g., changed their movement speed in response to feedback cues). One alternative to the current approach is using machine learning [111–113]. While machine learning models may offer a high degree of accuracy with respect to repetition counting [113], it may be at the expense of providing actionable feedback to the user to optimize movement performance. Approaches that enable a greater degree of child-specific customization in performance may be needed to accommodate different abilities and movement patterns (e.g., having customizable criteria that are matched to each child’s individual therapeutic needs and abilities and based on PT input). Partnering again with PT knowledge holders to guide the decisions on these changes will be essential. Lastly, tracking accuracy was also dependent on operating conditions. Families did not always follow recommendations made to optimize tracking performance. While these issues may be addressed through appropriate training and onboarding procedures (which need to be revised in collaboration with knowledge holders), it may also be necessary to add system checks to monitor operating conditions (e.g., proximity of the child to the sensor). Once technical and operational issues and limitations to the tracking/feedback have been addressed, an additional stage of usability testing with a small sample of participants will be required to ensure that implemented solutions work as intended, with the mixed methods SCED approach outlined in this paper valuable to guide this continued home-based evaluation.

## Conclusion

This study provided preliminary evidence that movement-tracking feedback within an ICP-HEP, Bootle Boot Camp, may support children’s behavioural engagement (i.e., exercise adherence and exercise fidelity), while having mixed effects on affective and cognitive engagement. While parents perceived feedback to be valuable, technical issues led to periods of disengagement for all participants. Children and parents felt positively overall about using the game for home exercise as compared to conventional HEPs. Game refinements and areas for future development (e.g., exercise progressions) are needed to optimize user experiences, with the mixed methods home use experience data from this study guiding these refinements. Future larger-scale evaluation will be needed to fully understand the impact of feedback on children’s behavioural, affective and cognitive engagement within an ICP-HEP to help inform future development and implementation of these technologies.

## Supplementary Information

Below is the link to the electronic supplementary material.

Supplementary Material 1

Supplementary Material 2

Supplementary Material 3

Supplementary Material 4

Supplementary Material 5

Supplementary Material 6

Supplementary Material 7

Supplementary Material 8

Supplementary Material 9

## Data Availability

The summarized datasets supporting the conclusions of this article, as they relate to exercise adherence, exercise fidelity, level of fun, and helpfulness ratings, are included within the article and its additional files. Additional data may be available from the corresponding author upon reasonable request.
