# Supplementary material for: Lessons learned while exploring the impact of movement-tracking feedback on the experiences of children with neuromotor disorders taking part in interactive home exercise programs: a multi-case mixed methods study
Source: J Neuroeng Rehabil. 2026 Feb 27;23:110. doi: 10.1186/s12984-025-01819-1 (PMC13040853; doi:10.1186/s12984-025-01819-1)
Supplement: Supplementary file 1 — Supplementary Material 1 [file 12984_2025_1819_MOESM1_ESM.docx]

**Appendix 1.** Individualized home exercise programs prescribed to each child participant by their treating physiotherapist.

| **CHILD 01**^+^ | | | |
| --- | --- | --- | --- |
| **Exercises** | **Repetitions or Timed Durations** | **Sets** | **Side (Right/Left/Bilateral)** |
| Hip flexion | 10 repetitions | 2 | Bilateral |
| Lateral step | 10 repetitions | 2 | Bilateral |
| Hip abduction | 10 repetitions | 2 | Bilateral |
| Squat | 10 repetitions | 2 | Bilateral |
| Kick | 10 repetitions | 2 | Bilateral |
| Backwards stepping | 10 repetitions | 2 | Bilateral |
| Single leg stance | 10 seconds | 3 | Bilateral |
| **CHILD 02** | | | |
| **Exercises** | **Repetitions or Timed Durations** | **Sets** | **Side (Right/Left/Bilateral)** |
| Sit to stand | 10 repetitions | 1 | Bilateral |
| Backwards stepping | 5 repetitions | 1 | Bilateral |
| Forward step | 30 seconds | 1 | Bilateral |
| Calf stretch | 30 seconds | 1 | Bilateral |
| Hamstring stretch | 60 seconds | 1 | Bilateral |
| **CHILD 03**^++^ | | | |
| **Exercises** | **Repetitions or Timed Durations** | **Sets** | **Side (Right/Left/Bilateral)** |
| Kick | 10 repetitions | 2 | Left |
| Sit to stand | 10 repetitions | 2 | Bilateral |
| Seated star jump | 10 repetitions | 2 | Bilateral |
| Lateral step | 10 repetitions | 2 | Bilateral |
| Tandem stance | 10 seconds | 3 | Bilateral |
| Forward step | 10 seconds | 3 | Bilateral |

^+^Child 01 had the home program reduced to one set for all exercises during week 2, with the full program reinstated during week 4, in consultation with his physiotherapist.

^++^Child 03 had the home program reduced to one set for all exercises after the first session, in consultation with her physiotherapist.
