# Supplementary material for: Lessons learned while exploring the impact of movement-tracking feedback on the experiences of children with neuromotor disorders taking part in interactive home exercise programs: a multi-case mixed methods study
Source: J Neuroeng Rehabil. 2026 Feb 27;23:110. doi: 10.1186/s12984-025-01819-1 (PMC13040853; doi:10.1186/s12984-025-01819-1)
Supplement: Supplementary file 2 — Supplementary Material 2 [file 12984_2025_1819_MOESM2_ESM.docx]

**Appendix 2.** Excerpt from interview guide demonstrating building of qualitative questions based on quantitative results.

| **Construct** | **Applicable Quantitative Findings** | **Qualitative Interview Questions** | **Rationale** |
| --- | --- | --- | --- |
| **Behavioural Engagement** | **Child 02:**  Completed 3 of 4 exercise sessions in weeks 3 and 5 (as compared to 1 session in all other weeks).  **Child 03:**  Highest exercise adherence achieved during session 3 (no-feedback condition). | “[Child 02] completed the most exercise sessions during weeks 3 and 5. Was something different about these particular weeks as compared to the other weeks?”  “Based on the system tracked data, you did the most exercise repetitions during the first week when you played the no-feedback version of the game. During this session, you did many more repetitions that what was prescribed to you. Can you explain what happened during this session? Were you counting the number of times you were doing an exercise on no-feedback days?” | To understand whether game features or environmental factors impacted behavioural engagement outcomes observed. |
| **Affective Engagement** | **Child 03:**  Median perceived level of fun rated 5 for both feedback and non-feedback versions of the game.  **Child 01:**  Selected the no-feedback version as being the most fun to play in the Bootle Boot Camp Acceptability Survey.  Awarded a 5-star rating to the no-feedback version of the game versus a 2-star rating for the feedback version of the game. | “Based on your level of fun rating scores, you appeared to have the same amount of fun every session. Did you find one version of the game more fun to play or were both versions the same?”  “You indicated that the no feedback version was the most fun to play in the survey. Can you tell me why it is the most fun version to play”  “You gave the no-feedback version a 5-star rating and the feedback version a 2-star rating. Can you tell me more about why you gave these two different ratings and why you felt this way?” | To confirm whether smiley face rating scores and survey responses accurately reflected child participants’ feelings towards the game versions and to understand why participants selected these responses. |
| **Cognitive Engagement** | **Child 03**:  Median perceived level of helpfulness for the body rated 5 for both feedback and no-feedback versions of the game. | “Based on your helpfulness rating scores, all exercise sessions with and without feedback were equally helpful for your body. Do you agree with this or feel differently?” | To confirm whether smiley face rating scores accurately reflected child participants’ feelings towards the game versions. |
