# Supplementary material for: Lessons learned while exploring the impact of movement-tracking feedback on the experiences of children with neuromotor disorders taking part in interactive home exercise programs: a multi-case mixed methods study
Source: J Neuroeng Rehabil. 2026 Feb 27;23:110. doi: 10.1186/s12984-025-01819-1 (PMC13040853; doi:10.1186/s12984-025-01819-1)
Supplement: Supplementary file 3 — Supplementary Material 3 [file 12984_2025_1819_MOESM3_ESM.docx]

**Appendix 3.** Codebook sample.

| **Category** | **Subcategory** | **Code** | **Code Definition** | **Exemplar Quote** |
| --- | --- | --- | --- | --- |
| **Cognitive Engagement** | Perceived benefits and value | Goal setting | Related to the process of setting therapeutic goals. | “Do you feel like it accomplished that goal for you – that it was able to get him up and moving?” [Interviewer]  “Yes.” [Parent 01] |
|  |  | Achievement | Comments made describing the child’s completion of goals or tasks they set out to do. | “Our hope was to get her to do the most basic exercises at home more than you know a few times a week. So we're really grateful that we accomplished that.”  [Parent 03] |
|  |  | Expectations | Beliefs about how useful the program would be in enacting desired change. | “I thought both [versions of the game] would be the same.” [Parent 01] |
|  |  | Intervention efficacy | Beliefs about how useful the program was in achieving desired change. | “And so you attribute her success for those goals, to, rather in part to playing the game?” [Interviewer]  “Not in part. I think it’s directly related to the game.” [Parent 03] |
|  |  | Helpful | Beliefs about the usefulness of the game or specific game features for home exercise practice. | “They teach you how to do the exercises.” [Child 01]  “I think the Coach is helpful.” [Parent 01] |
|  |  | Physical activity | Beliefs about the use of Bootle Boot Camp to promote movement and physical activity. | “At least he moves. Otherwise, he just sits down and watches YouTube and doesn’t exercise at all.”  [Parent 01] |
|  |  | Motor memory | Beliefs about the impact of repetitive movement practice. | “I did anticipate that things like sit to stand and what not would become a bit easier. A lot too because of so many repetitions, it’s then you know the motor memory.” [Parent 02] |
