# Supplementary material for: Lessons learned while exploring the impact of movement-tracking feedback on the experiences of children with neuromotor disorders taking part in interactive home exercise programs: a multi-case mixed methods study
Source: J Neuroeng Rehabil. 2026 Feb 27;23:110. doi: 10.1186/s12984-025-01819-1 (PMC13040853; doi:10.1186/s12984-025-01819-1)
Supplement: Supplementary file 5 — Supplementary Material 5 [file 12984_2025_1819_MOESM5_ESM.docx]

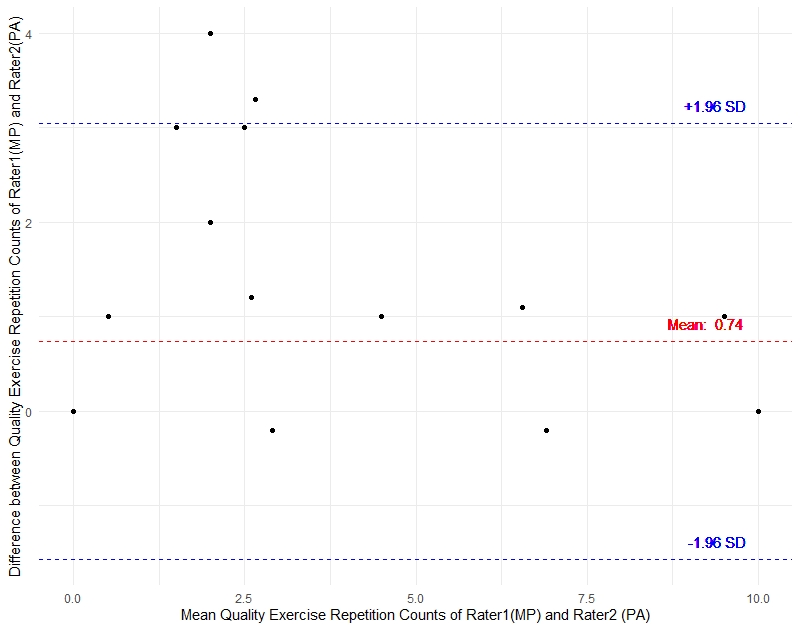


**Appendix 5.** Bland-Altman plot of acceptable exercise repetition counts (exercise fidelity) between rater MP and rater PA.
