# Supplementary material for: Lessons learned while exploring the impact of movement-tracking feedback on the experiences of children with neuromotor disorders taking part in interactive home exercise programs: a multi-case mixed methods study
Source: J Neuroeng Rehabil. 2026 Feb 27;23:110. doi: 10.1186/s12984-025-01819-1 (PMC13040853; doi:10.1186/s12984-025-01819-1)
Supplement: Supplementary file 6 — Supplementary Material 6 [file 12984_2025_1819_MOESM6_ESM.docx]

**Appendix 6.** Proportion of prescribed exercise repetitions completed with acceptable form (i.e. exercise fidelity) across sessions and individual exercises for child 01.

| **COMPARISON PHASE** | | | | | | | | | | | | | | | | **BEST ALONE (FEEDBACK) ^+^** | | | | | | | |
| --- | --- | --- | --- | --- | --- | --- | --- | --- | --- | --- | --- | --- | --- | --- | --- | --- | --- | --- | --- | --- | --- | --- | --- |
| **Week 1** | | | | **Week 2** | | | | **Week 3** | | | | **Week 4** | | | | **Week 5** | | | | **Week 6** | | | |
| **1** | **2** | **3** | **4** | **5** | **6** | **7** | **8** | **9** | **10** | **11** | **12** | **13** | **14** | **15** | **16** | **17** | **18** | **19** | **20** | **21** | **22** | **23** | **24** |
| NF | NF | F | NF | * | * | NF | F | F | NF | F | NF | * | NF | F | NF | F | F | F | * | F* | F | * | F |
| **Hip Flexion** | | | | | | | | | | | | | | | | | | | | | | | |
| 0.38 | 0.12 | 0.55 | - | - | - | 0.15 | 0.05 | 0.50 | 0.00 | 0.10 | 0.15 | - | 0.00 | 0.05 | 0.05 | 0.55 | 0.60 | 0.78 | - | 0.70 | 0.73 | - | 0.78 |
| **Lateral Step** | | | | | | | | | | | | | | | | | | | | | | | |
| 0.45 | 0.10 | 0.50 | 0.54 | - | - | 0.42 | 0.45 | 0.20 | 0.15 | 0.40 | 0.15 | - | 0.40 | 0.32 | 0.35 | 0.55 | 0.42 | 0.52 | - | 0.50 | 0.85 | - | 0.50 |
| **Hip Abduction** | | | | | | | | | | | | | | | | | | | | | | | |
| 0.25 | 0.05 | 0.15 | 0.06 | - | - | - | 0.25 | 0.50 | 0.25 | 0.15 | 0.70 | - | 0.28 | 0.10 | 0.48 | 0.30 | 0.25 | 0.50 | - | 0.25 | 0.15 | - | 0.15 |
| **Squat** | | | | | | | | | | | | | | | | | | | | | | | |
| 0.83 | 0.10 | 0.35 | 0.00 | - | - | 0.70 | 0.50 | 0.30 | 0.70 | 0.40 | 0.80 | - | 0.40 | 0.15 | 0.40 | 0.40 | 0.15 | 0.20 | - | 0.60 | 0.15 | - | 0.20 |
| **Kicking** | | | | | | | | | | | | | | | | | | | | | | | |
| 0.21 | 0.32 | 0.25 | - | - | - | 0.20 | 0.20 | 0.25 | 0.25 | 0.20 | 0.35 | - | 0.30 | 0.12 | 0.40 | 0.28 | 0.10 | 0.28 | - | 0.12 | 0.05 | - | 0.28 |
| **Backwards stepping** | | | | | | | | | | | | | | | | | | | | | | | |
| 0.33 | 0.28 | 0.75 | - | - | - | 0.32 | 0.50 | 0.35 | 0.20 | 0.45 | 0.25 | - | 0.18 | 0.45 | 0.35 | 0.50 | 0.45 | 0.40 | - | 0.40 | 0.43 | - | 0.20 |
| **Single leg stance** | | | | | | | | | | | | | | | | | | | | | | | |
| 0.33 | 0.24 | 0.52 | - | - | - | 0.35 | 0.23 | 0.36 | 0.36 | 0.93 | 0.36 | - | 0.33 | 0.41 | 0.34 | 0.33 | 0.45 | 0.52 | - | - | 0.51 | - | 0.51 |
| **OVERALL (MEAN) EXERCISE FIDELITY BY SESSION** | | | | | | | | | | | | | | | | | | | | | | | |
| **0.39** | **0.18** | **0.44** | **0.16** | **-** | **-** | **0.38** | **0.31** | **0.35** | **0.27** | **0.38** | **0.39** | **-** | **0.27** | **0.24** | **0.34** | **0.42** | **0.35** | **0.46** | **-** | **0.38** | **0.41** | **-** | **0.34** |

F=feedback, N=no feedback

^+^Game version in best-alone phase (weeks 5 and 6) determined by highest mean proportion of prescribed exercise repetitions.

*Indicates that technical issues were experienced.

- indicates that no exercise data is available (e.g., exercise was skipped, no video data available for review, technical limitation).
