# Supplementary material for: Lessons learned while exploring the impact of movement-tracking feedback on the experiences of children with neuromotor disorders taking part in interactive home exercise programs: a multi-case mixed methods study
Source: J Neuroeng Rehabil. 2026 Feb 27;23:110. doi: 10.1186/s12984-025-01819-1 (PMC13040853; doi:10.1186/s12984-025-01819-1)
Supplement: Supplementary file 7 — Supplementary Material 7 [file 12984_2025_1819_MOESM7_ESM.docx]

**Appendix 7**. Selection of Bootle Boot Camp Acceptability survey results for child participants 01 and 03, as rated on customized 5-point Likert rating scales where 1=disagree and 5=agree. No survey was completed by child 02.

| **SURVEY QUESTION** | **CHILD 01** | **CHILD 03** |
| --- | --- | --- |
| **Botley’s Coaching and Feedback (/5 where 1=disagree and 5=agree)** | | |
| Botley’s coaching and feedback helped me do the exercises better. | 1 | 5 |
| Botley’s coaching and feedback made me want to exercise more. | 2 | 3 |
| Botley’s coaching and feedback helped me learn new exercises. | 2 | 5 |
| ^a^Botley’s coaching and feedback was not frustrating. | 2 | 5 |
| ^a^There was just enough coaching and feedback. | 1 | 5 |
| Botley’s coaching and feedback was motivating. | 1 | 5 |
| ^a^Botley’s coaching and feedback was not distracting. | 2 | 5 |
| I liked getting feedback about how I did the exercises. | 1 | 5 |
| **Preferred Version of the Game** | | |
| The version that was the most fun to play. | WITHOUT Coach Botley | Both versions were the SAME |
| The version that helped my body the most. | WITHOUT Coach Botley | Both versions were the SAME |
| The version that helped me try and reach my goals the most. | WITHOUT Coach Botley | Both versions were the SAME |
| The version that helped me get stronger. | WITHOUT Coach Botley | Both versions were the SAME |
| The version that helped me with my balance. | WITHOUT Coach Botley | Both versions were the SAME |
| The version that helped me become more flexible. | WITHOUT Coach Botley | Both versions were the SAME |
| The version that helped me train my body for longer. | WITHOUT Coach Botley | Both versions were the SAME |
| The version that made me feel the most confident that I could do the exercises well. | WITHOUT Coach Botley | Both versions were the SAME |
| **Overall Play Experience** | | |
| What star rating would you give Bootle Boot Camp WITHOUT Coach Botley (no movement feedback)? | 5 | 1 |
| What star rating would you give Bootle Boot Camp WITH Coach Botley (movement feedback)? | 2 | 5 |

^a^denotes items that were stated in negative wording in the original survey. Items have been stated in positive wording with reverse-scoring shown to maintain consistency in scoring procedure where 1=disagree, 2=somewhat disagree, 3=neither agree nor disagree, 4=somewhat agree, and 5=agree.

Note: no survey data is available for child 02 as she opted to not complete the survey.
