# Supplementary material for: Lessons learned while exploring the impact of movement-tracking feedback on the experiences of children with neuromotor disorders taking part in interactive home exercise programs: a multi-case mixed methods study
Source: J Neuroeng Rehabil. 2026 Feb 27;23:110. doi: 10.1186/s12984-025-01819-1 (PMC13040853; doi:10.1186/s12984-025-01819-1)
Supplement: Supplementary file 8 — Supplementary Material 8 [file 12984_2025_1819_MOESM8_ESM.docx]

**Appendix 8.** Proportion of prescribed exercise repetitions completed with acceptable form (i.e. exercise fidelity) across sessions and individual exercises for child 02.

| **COMPARISON PHASE** | | | | | | | | | | | | | | | | **BEST ALONE (NO FEEDBACK) ^+^** | | | | | | | |
| --- | --- | --- | --- | --- | --- | --- | --- | --- | --- | --- | --- | --- | --- | --- | --- | --- | --- | --- | --- | --- | --- | --- | --- |
| **Week 1** | | | | **Week 2** | | | | **Week 3** | | | | **Week 4** | | | | **Week 5** | | | | **Week 6** | | | |
| **1** | **2** | **3** | **4** | **5** | **6** | **7** | **8** | **9** | **10** | **11** | **12** | **13** | **14** | **15** | **16** | **17** | **18** | **19** | **20** | **21** | **22** | **23** | **24** |
| F |  |  |  | F |  |  |  | NF | F | F |  | NF |  |  |  | NF | NF | NF |  | NF |  |  |  |
| **Sit to Stand** | | | | | | | | | | | | | | | | | | | | | | | |
| 0.00 | - | - | - | 0.00 | - | - | - | 0.00 | 0.00 | 0.00 | - | 0.00 | - | - | - | 0.00 | 0.00 | 0.00 | - | 0.00 | - | - | - |
| **Backwards Stepping** | | | | | | | | | | | | | | | | | | | | | | | |
| 0.10 | - | - | - | 0.30 | - | - | - | 0.00 | 0.00 | 0.10 | - | 0.00 | - | - | - | 0.00 | 0.20 | 0.20 | - | 0.20 | - | - | - |
| **Forward Step** | | | | | | | | | | | | | | | | | | | | | | | |
| 1.00 | - | - | - | 0.81 | - | - | - | 0.53 | 0.05 | 0.88 | - | 0.81 | - | - | - | 0.91 | 0.90 | 0.84 | - | 0.90 | - | - | - |
| **Calf Stretch** | | | | | | | | | | | | | | | | | | | | | | | |
| 0.00 | - | - | - | 0.52 | - | - | - | 0.00 | 0.63 | 0.99 | - | 0.00 | - | - | - | 0.14 | 0.00 | 0.15 | - | 0.00 | - | - | - |
| **Hamstring Stretch** | | | | | | | | | | | | | | | | | | | | | | | |
| 0.08 | - | - | - | 1.00 | - | - | - | 0.66 | 0.24 | 0.15 | - | 0.99 | - | - | - | 0.78 | 0.94 | 0.73 | - | 0.28 | - | - | - |
| **OVERALL (MEAN) EXERCISE FIDELITY BY SESSION** | | | | | | | | | | | | | | | | | | | | | | | |
| **0.24** | **-** | **-** | **-** | **0.54** | **-** | **-** | **-** | **0.24** | **0.18** | **0.42** | **-** | **0.36** | **-** | **-** | **-** | **0.37** | **0.41** | **0.38** | **-** | **0.28** | **-** | **-** | **-** |

F=feedback, N=no feedback

^+^Game version in best-alone phase (weeks 5 and 6) determined by highest mean proportion of prescribed exercise repetitions.

Blank cells indicate exercise sessions that were missed by the child for unknown reasons.

- indicates that no exercise data is available (e.g., exercise was skipped, no video data available for review, technical limitation).
