# Supplementary material for: Lessons learned while exploring the impact of movement-tracking feedback on the experiences of children with neuromotor disorders taking part in interactive home exercise programs: a multi-case mixed methods study
Source: J Neuroeng Rehabil. 2026 Feb 27;23:110. doi: 10.1186/s12984-025-01819-1 (PMC13040853; doi:10.1186/s12984-025-01819-1)
Supplement: Supplementary file 9 — Supplementary Material 9 [file 12984_2025_1819_MOESM9_ESM.docx]

**Appendix 9.** Proportion of prescribed exercise repetitions completed with acceptable form (i.e. exercise fidelity) across sessions and individual exercises for child 03.

| **COMPARISON PHASE** | | | | | | | | | | | | | | | | **BEST ALONE (FEEDBACK)^+^** | | | | | | | |
| --- | --- | --- | --- | --- | --- | --- | --- | --- | --- | --- | --- | --- | --- | --- | --- | --- | --- | --- | --- | --- | --- | --- | --- |
| **Week 1** | | | | **Week 2** | | | | **Week 3** | | | | **Week 4** | | | | **Week 5** | | | | **Week 6** | | | |
| **1** | **2** | **3** | **4** | **5** | **6** | **7** | **8** | **9** | **10** | **11** | **12** | **13** | **14** | **15** | **16** | **17** | **18** | **19** | **20** | **21** | **22** | **23** | **24** |
| F | NF | NF | F | F | NF* | NF* |  | F |  |  |  | NF^ | F^ | NF^ | F | F | NF |  |  | F | F | * | * |
| **Kick** | | | | | | | | | | | | | | | | | | | | | | | |
| 0.00 | 0.00 | 0.00 | 1.00 | 0.00 | 0.30 | - | - | 0.00 | - | - | - | 0.10 | 0.60 | 0.30 | 0.00 | 0.00 | 0.00 | - | - | 0.00 | 0.00 | - | - |
| **Sit to Stand** | | | | | | | | | | | | | | | | | | | | | | | |
| 0.35 | - | 0.00 | 0.00 | 0.30 | 0.10 | - | - | 0.60 | - | - | - | 0.50 | 1.30 | 0.40 | 0.90 | 0.80 | 0.10 | - | - | 0.60 | 0.10 | - | - |
| **Seated Star Jump** | | | | | | | | | | | | | | | | | | | | | | | |
| 0.10 | 0.00 | 0.30 | 0.20 | 0.50 | 0.20 | 0.40 | - | 0.60 | - | - | - | 1.00 | 0.60 | 0.10 | 1.20 | 0.70 | 0.50 | - | - | 0.40 | 0.90 | - | - |
| **Lateral Step** | | | | | | | | | | | | | | | | | | | | | | | |
| 0.00 | - | 0.10 | 0.10 | 0.00 | 0.00 | 0.0 | - | 0.00 | - | - | - | 0.00 | 0.60 | 0.55 | 0.95 | 0.75 | - | - | - | 0.30 | 0.85 | - | - |
| **Tandem Stance** | | | | | | | | | | | | | | | | | | | | | | | |
| 1.50 | - | 0.58 | 0.86 | 0.62 | 0.62 | 0.25 | - | 1.00 | - | - | - | 0.04 | 1.14 | 0.45 | 1.40 | 1.15 | 0.58 | - | - | 0.81 | 0.44 | - | - |
| **Forward Step** | | | | | | | | | | | | | | | | | | | | | | | |
| 0.72 | 0.76 | 0.48 | 0.62 | 0.72 | 0.66 | - | - | 1.25 | - | - | - | 0.43 | 0.83 | 0.46 | 0.64 | 1.14 | 0.58 | - | - | 1.30 | 1.00 | - | - |
| **OVERALL (MEAN) EXERCISE FIDELITY PER SESSION** | | | | | | | | | | | | | | | | | | | | | | | |
| **0.68** | **0.38** | **0.32** | **0.53** | **0.60** | **0.45** | **0.22** | **-** | **0.71** | **-** | **-** | **-** | **0.32** | **0.84** | **0.40** | **0.89** | **0.85** | **0.42** | **-** | **-** | **0.69** | **0.59** | **-** | **-** |

F=feedback, N=no feedback

^+^Game version in best-alone phase (weeks 5 and 6) determined by highest mean proportion of prescribed exercise repetitions.

*Indicates that technical issues were experienced.

Blank cells indicate exercise sessions that were missed by the child for unknown reasons.

^ indicates multiplayer sessions.

- indicates that no exercise data is available (e.g., exercise was skipped, no video data available for review, technical limitation).
